# Duodenal dysbiosis is linked to altered ferroportin related transcriptomics programs in iron deficiency anemia

**DOI:** 10.3389/fnut.2026.1836940

**Published:** 2026-07-01

**Authors:** Siwani Agrawal, Rishikesh Dash, Pranav Jumin, Subash Chandra Samal, Shaktiprasad Mishra, Sunil Kumar Raghav, Balakrishnan S. Ramakrishna, Balamurugan Ramadass

**Affiliations:** 1 Department of Biochemistry, All India Institute of Medical Sciences, Bhubaneswar, Odisha, India; 2 Department of Gastroenterology, All India Institute of Medical Sciences, Bhubaneswar, Odisha, India; 3 Institute of Life Sciences, Autonomous Institute Under Department of Biotechnology, Government of India, Bhubaneswar, Odisha, India; 4 Institute of Gastroenterology, SRM Institutes of Medical Science, Chennai, India; 5 Centre of Excellence for Clinical Microbiome and Research (CCMR), All India Institute of Medical Sciences, Bhubaneswar, Odisha, India; 6 Adelaide Medical School, Faculty of Health and Medical Sciences, The University of Adelaide, Adelaide, SA, Australia

**Keywords:** duodenal microbiome, enterocyte iron trap, ferroportin, hepcidin, iron deficiency anemia, long non-coding RNA, Th17 inflammation

## Abstract

**Background & Aims:**

Iron deficiency anemia (IDA) affects over two billion people, yet up to half of patients show inadequate response to oral iron therapy. We hypothesized that IDA is a primary duodenal mucosal disorder where dysbiosis and immune polarization converge to impair enterocyte iron export. This study integrates mucosal-associated microbiome and transcriptomic profiling to elucidate mechanisms underlying impaired iron handling.

**Methods:**

Duodenal biopsies from women with IDA (*n* = 11) and matched controls (*n* = 9) underwent paired 16S rRNA and RNA-Seq. A Microbial Redox Index (MRI) quantified oxygen-tolerant taxa. Multilayer network modeling linked microbial hubs to epithelial transcriptional remodeling in iron-handling, inflammatory, and barrier-integrity pathways.

**Results:**

IDA subjects demonstrated expected hematological deficits (hemoglobin 10.02 ± 0.82 vs. 12.69 ± 0.67 g/dL; ferritin 10.7 [8.2–35.3] vs. 49.7 [28.4–58.7] ng/mL; *P* < 0.05). Although the overall ratio of oxygen-tolerant to anaerobic taxa was comparable between groups (*P* = 0.44), IDA was marked by a collapse of homeostatic ecological control. In controls, Group V a/V b anaerobes showed a strong inverse correlation with Shannon diversity (*P* = 0.009), indicating a stable, niche-restricting anaerobic core. This relationship was lost in IDA, where both oxygen-tolerant and anaerobic taxa displayed positive correlations with Th17 skewed inflammation (IL17A log2FC = +3.59), hypoxic stress (EGLN3 log2FC = +1.31), and sensitized BMP signaling (BMPR2 log2FC = +0.50). These transcriptomic signatures could reflect a functional ferroportin blockade, as reflected by SLC40A1 mRNA upregulation (log2FC = +1.02) concurrent with a proposed model of post translational ferroportin suppression, despite profound cellular iron starvation (TFRC log2FC = +1.58; SLC11A2 log2FC = +2.2). Together, these features are consistent with a possible enterocyte iron retention phenotype. The lncRNA LOC124902620 emerged as a central regulatory hub linking dysbiosis to iron-handling genes.

**Conclusions:**

IDA is a duodenal mucosal disorder where dysbiosis-driven redox shifts and immune activation could support a model of hepcidin associated ferroportin downregulation. This is consistent with a proposed enterocyte iron retention phenotype. Microbial hubs and the LOC124902620 axis are promising targets for precision interventions to restore mucosal iron export.

## Graphical Abstract

**Figure:**
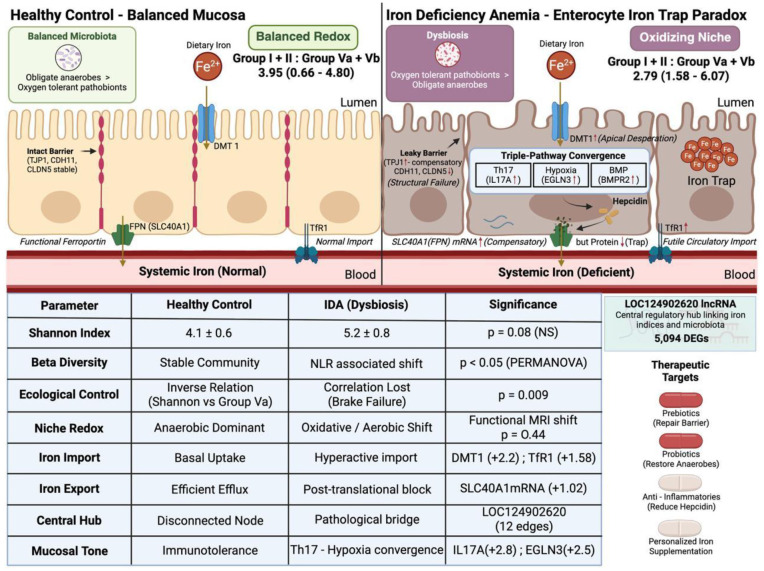
Duodenal dysbiosis in women with iron deficiency anemia disrupts mucosal ecological control (P=0.009), triggering a triple-pathway convergence Th17 inflammation (IL17A), hypoxic signaling (EGLN3), and BMP signaling (BMPR2) that could contribute to post translational ferroportin suppression (SLC40A1) and functional basolateral blockade. A possible outcome is an enterocyte iron retention phenotype: increased apical iron import signals (TFRC↑, DMT1↑) with impaired iron efflux, a phenotype coordinated by the lncRNA LOC124902620

## Introduction

Iron deficiency anemia (IDA) is the most common nutritional disorder worldwide, affecting more than two billion individuals and accounting for nearly half of all anemia cases. Despite the widespread availability of oral iron supplements, 30–50% of patients fail to normalize hemoglobin levels (1), and many experience gastrointestinal intolerance that limits adherence (2). These high rates of treatment failure indicate that models focused solely on systemic iron depletion are insufficient to explain the underlying biology of IDA (3, 4). This discrepancy has prompted growing recognition that IDA may arise not only from inadequate iron supply but also from defects within the duodenal mucosa, where iron absorption is initiated and regulated.

The duodenum is the primary and rate-limiting site of dietary iron absorption, integrating luminal substrates, microbial metabolites, mucosal immunity, and epithelial transport machinery to maintain systemic iron homeostasis. Iron uptake requires coordinated apical import via divalent metal transporter-1 (DMT1) and basolateral export via ferroportin, the sole mammalian iron exporter (5). Ferroportin is exquisitely sensitive to inflammatory and microbial cues through hepcidin-mediated internalization and degradation, positioning the duodenal epithelium as a critical control point for iron bioavailability (6). Disruption of ferroportin function results in intracellular iron retention and ineffective absorption, a phenomenon increasingly suspected in patients who fail oral iron therapy (7, 8).

The mucosa-associated microbiome is emerging as a key regulator of epithelial iron handling (9). Microbes compete for luminal iron through siderophores (10), modulate pH and redox potential, which determine the Fe^2+^/Fe^2+^ balance, and influence host iron-responsive genes through metabolite and cytokine induction (11). Yet, most microbiome–iron studies rely on stool samples (12), which reflect colonic rather than duodenal communities. The duodenum harbors a distinct, oxygen-tolerant microbial ecosystem shaped by gastric oxygen exposure, bile acids, pancreatic enzymes, and rapid nutrient flow. These microbes reside in direct proximity to the brush border where iron transporters are expressed, making them uniquely positioned to modulate epithelial iron trafficking (13).

The duodenal mucosa is also immunologically active, with enrichment of Th17 cells, IgA-producing plasma cells, and dendritic cells (14). Dysbiosis-driven immune activation can induce IL-17A, IL-6, and IL-1β, promoting hepatic hepcidin production and suppressing ferroportin-mediated iron export (15). In parallel, inflammation-associated barrier disruption permits microbial antigens to penetrate the mucosa, amplifying immune activation in a feed-forward loop (16). Together, these processes suggest that duodenal dysbiosis and mucosal inflammation may converge to impair ferroportin-dependent iron trafficking, yet this has never been systematically examined in humans (17).

To date, no study has integrated duodenal microbiome profiling, mucosal transcriptomics, and iron-regulatory gene networks to elucidate the molecular basis of IDA at the site of absorption. The specific microbial taxa, immune pathways, and epithelial circuits that coordinate the dysbiosis–inflammation–hepcidin–ferroportin axis remain unknown. Here, we present the first multiomic investigation of the duodenal mucosa in IDA, combining 16S rRNA sequencing, RNA-Seq transcriptomics, and integrative network modeling in a well-characterized cohort of young women with IDA and matched controls. We introduce a Microbial Redox Index (MRI) to quantify shifts in microbial oxygen tolerance and investigate how duodenal dysbiosis, mucosal immune remodeling, and epithelial iron-handling pathways may converge to impair ferroportin-mediated iron export and contribute to mucosal iron retention.These findings provide a mechanistic framework for understanding oral iron treatment failure and identify microbial and epithelial targets for restoring mucosal iron export.

## Materials and methods

### Study design and ethical approval

This observational, proof-of-concept study was conducted at the All India Institute of Medical Sciences (AIIMS), Bhubaneswar, India. Female patients aged 18–40 years presenting with dyspepsia and scheduled for upper gastrointestinal endoscopy were prospectively screened between January 2022 and December 2023. The study was approved by the Institutional Ethics Committee of AIIMS Bhubaneswar (IEC/AIIMS BBSR/PhD Thesis/2023-24/02), and written informed consent was obtained from all participants in accordance with the Declaration of Helsinki.

### Study population

**Figure 1:**
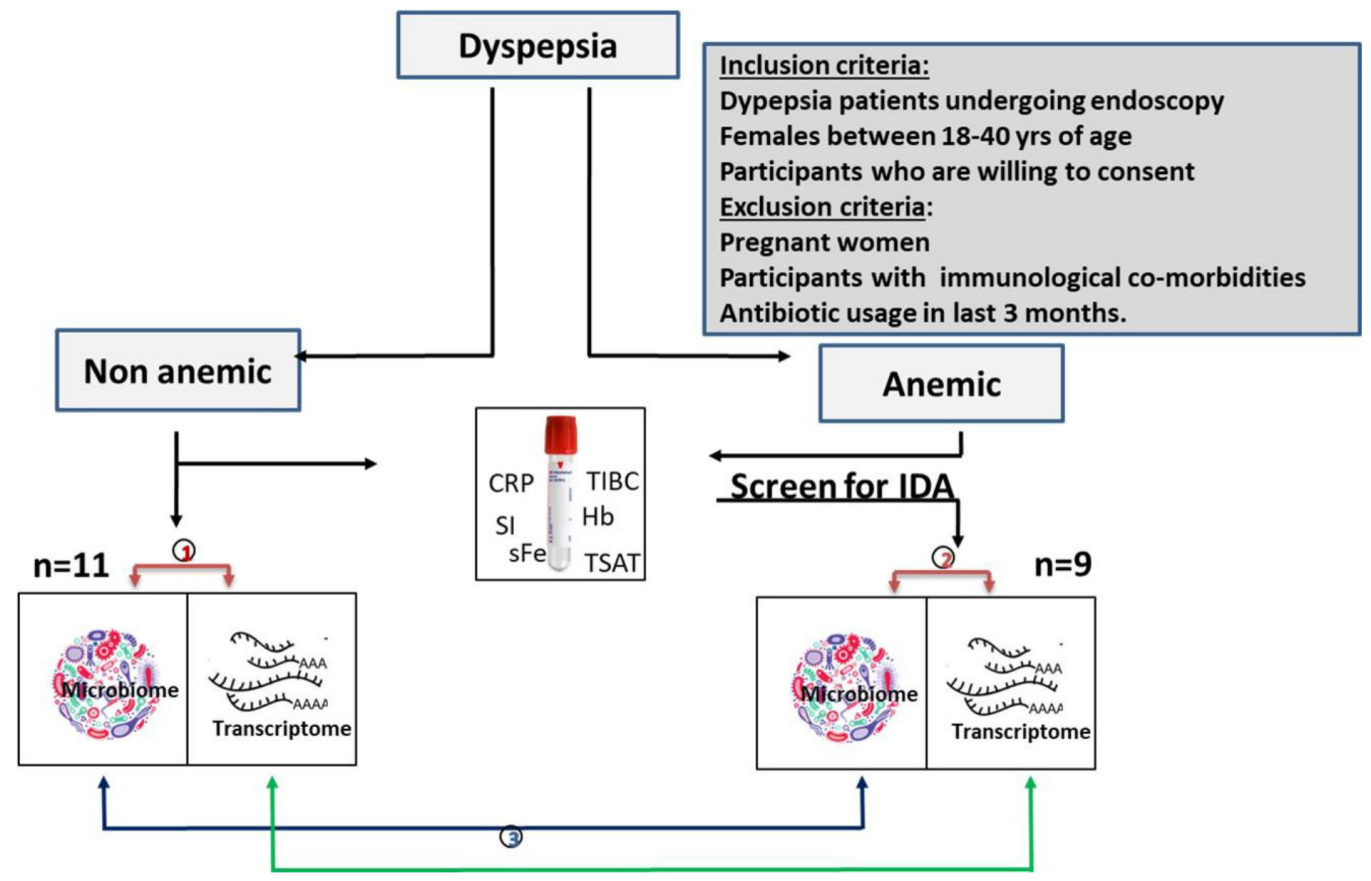
Study design and multi-omic workflow. Overview of participant recruitment, duodenal biopsy collection, and integrated analytical pipeline, including mucosa-associated microbiome profiling, RNASeq transcriptomics, and clinical iron indices.

Consecutive women with dyspepsia aged 18–40 years undergoing diagnostic upper gastrointestinal endoscopy at the Department of Gastroenterology, AIIMS Bhubaneswar, willing to provide duodenal biopsies for research purposes were recruited (Figure 1). Participants were stratified into iron-deficiency anemia (IDA) and control groups based on World Health Organization criteria: IDA defined as hemoglobin < 12 g/dL and serum ferritin < 15 ng/mL; controls defined as hemoglobin ≥ 12 g/dL and Ferritin ≥ 15 ng/mL. Exclusion criteria included pregnancy or lactation; chronic inflammatory diseases such as inflammatory bowel disease, celiac disease, or autoimmune disorders; active gastrointestinal bleeding or peptic ulcer disease; chronic kidney disease (eGFR < 60 mL/min/1.73 m^2^); chronic liver disease or cirrhosis (Child-Pugh B or C); active malignancy; blood transfusion within the preceding 3 months; antibiotic or probiotic use within 4 weeks; oral or intravenous iron supplementation within 4 weeks; immunosuppressive therapy (corticosteroids, biologics, disease-modifying antirheumatic drugs); HIV, hepatitis B, or hepatitis C infection; and prior gastric or duodenal surgery.

### Sample size calculation

This investigation was designed as a high-resolution, proof-of-concept discovery study employing an integrated multi-omic strategy. Given the exploratory nature of the work and the absence of prior duodenal mucosa–associated microbiome or host transcriptomic datasets in human IDA, a formal a priori power calculation for the microbiome component was not feasible. Instead, a targeted sample size of *n* = 10 per Group (final enrolment: *n* = 11 IDA, *n* = 9 Control) was selected based on established feasibility and statistical performance for deep sequencing transcriptomic studies. For RNA-Seq profiling (18), a cohort of this size provides >80% power to detect genes with an absolute log_2_ fold-change ≥ 1.5 at moderate expression levels (mean normalized counts > 50), which is appropriate for identifying the significant-effect of transcriptional perturbations expected in iron-handling, inflammatory, and epithelial stress-response pathways. Moreover, prior studies integrating mucosa-associated microbiome and host gene expression data demonstrate that similar cohort sizes are sufficient to resolve community-level microbial shifts and host–microbe interaction patterns. This sample size, therefore, balances statistical rigor with the practical constraints of obtaining high-quality duodenal biopsies for multi-omic analysis.

### Clinical data and sample collection

At enrollment, demographic characteristics (age, body mass index), menstrual history, dietary habits (vegetarian vs. non-vegetarian), medication use, and dyspepsia severity were recorded. After eligibility confirmation, peripheral blood and duodenal (D2) mucosal biopsy samples were obtained. Fasting venous blood (10 mL) was analyzed for complete blood count using the Sysmex XN-1000 (Sysmex Corporation, Kobe, Japan). Serum aliquots (500 μL) were processed on the AU5800 Beckman Coulter Chemistry Analyzer to quantify serum iron (SI), serum ferritin (sFe), unbound iron-binding capacity (UIBC), and C-reactive protein (CRP). To eliminate the confounding effects of systemic inflammation, a CRP value ≥ 4 mg/L was used as exclusion criteria. Total iron-binding capacity (TIBC) was calculated as SI + UIBC, and transferrin saturation (TSAT) as (SI / TIBC) × 100. Hematological profiling included hemoglobin concentration, white blood cell (WBC) count, differential leukocyte counts, and red cell indices, mean corpuscular volume (MCV), mean corpuscular hemoglobin (MCH), mean corpuscular hemoglobin concentration (MCHC), and red cell distribution width (RDW). Anemia was defined as hemoglobin < 12 g/dL, and participants were classified as iron-deficient anemic if they met any of the following biochemical criteria: TSAT < 16%, SI < 30 μg/L, sFe < 15 μg/L, or TIBC > 410 μg/dL. NLR (Neutrophil/Lymphocyte count ratio) was calculated that is used as a continuous marker of subclinical immune tone to examine its ecological association with duodenal microbial structure.

During upper gastrointestinal endoscopy (Olympus GIF-H190; Olympus Corporation, Tokyo, Japan), four mucosal biopsies were obtained from the second portion of the duodenum (D2), 2–3 cm distal to the ampulla of Vater, avoiding areas with visible inflammation, erosions, or ulceration. Biopsies were immediately partitioned for multiomic analysis: two were snap-frozen in liquid nitrogen and stored at −80 °C for 16S rRNA gene sequencing, while two were placed in RNAlater (Thermo Fisher Scientific, Waltham, MA), incubated overnight at 4 °C, and stored at −80 °C for RNA-Seq. Endoscopists were blinded to IDA status at the time of biopsy collection. Clinical, biochemical, and multiomic datasets were subsequently integrated to investigate disruption of the gut–iron–immune axis in IDA.

### DNA extraction and 16S rRNA gene sequencing

Approximately 1–3 mg of duodenal mucosal tissue was used for DNA extraction using the QIAamp PowerFecal Pro DNA Kit (QIAGEN) according to the manufacturer's protocol. The V3–V4 region of the bacterial 16S rRNA gene was amplified from 12.5 ng of DNA using primers 5′-AGAGTTTGATGMTGGCTCAG-3′ and 5′-TTACCGCGGCMGCSGGCAC-3′ containing Illumina adapter overhangs (19). PCR conditions were: 95 °C for 3 min; 25 cycles of 95 °C for 15 s, 60 °C for 15 s, and 72 °C for 2 min; followed by a final extension at 72 °C for 10 min. Amplicons were purified using AMPure XP beads, indexed with the Nextera XT Index Kit v2, and libraries that passed quality control (Agilent TapeStation) were sequenced on an Illumina NexSeq2000 platform (2 × 300 bp paired-end reads). Sequencing data are available under BioProject ID PRJNA1062572.

### RNA extraction and transcriptomic sequencing

Duodenal tissue (3–6 mg) was processed using the NucleoSpin RNA Plus Mini Kit (Macherey-Nagel). Tissue was homogenized with a sterile micropestle and passed through a 20-gauge needle. RNA was eluted in preheated nuclease-free water (65 °C) and assessed on an Agilent Bioanalyzer; samples with an RNA integrity number (RIN) > 7 were retained. For library preparation, 1 μg of total RNA was enriched for mRNA using the NEBNext Poly(A) mRNA Isolation Kit and converted into stranded mRNA libraries using the NEBNext Ultra II Directional RNA Library Prep Kit for Illumina (New England Biolabs), with indexing performed using NEBNext Oligo Kits. Libraries passing quality control (Bioanalyzer DNA1000 Chip) were sequenced at the Institute of Life Sciences, Bhubaneswar, on an Illumina NovaSeq 6000 platform using 2 × 150 bp paired-end reads, targeting 40 million reads per sample. Transcriptomic data are deposited under BioProject ID PRJNA1062572.

### Microbiome read processing and taxonomic profiling

Raw FASTQ files were quality-checked using FastQC and adapter-trimmed with Trim Galore v0.6.10 (20, 21). Paired-end reads were merged and filtered using VSEARCH v2.27.0. Taxonomic classification was performed using Kraken2 (22), yielding 2,489 operational taxonomic units (OTUs), including 1,960 species-level assignments. OTUs with < 50 total counts were removed, yielding 777 OTUs; the top 200 species, representing 86.7% of total abundance, were retained for downstream analyses.

Alpha diversity metrics (Observed OTUs, Simpson, Shannon,Pielou's evenness) and beta diversity (Bray–Curtis dissimilarity, PCoA) were computed using the vegan v2.6.4 package. (23) Group differences were assessed using the Wilcoxon rank-sum test, Adonis (10,000 permutations), and SIMPER analysis.

### Microbial redox index (MRI) calculation

To quantify shifts in microbial oxygen tolerance and infer the duodenal mucosal redox state, we developed a Microbial Redox Index (MRI) (24). All detected operational taxonomic units (OTUs) were classified into five redox-phenotype groups based on cytochrome oxidase and catalase gene content, using Bergey's Manual (25) and NCBI GenBank annotations. Group I comprised aerobic organisms possessing low-affinity cytochrome oxidases (e.g., Sphingomonas, Stenotrophomonas, Pseudomonas), whose growth indicates a highly oxygenated, high-redox mucosal environment. Group II included aerobes expressing only microaerobic cytochrome oxidases (e.g., Enterococcus hemoperoxidus, Streptococcus agalactiae, Rothia mucilaginosa), enabling growth under low-oxygen, moderately oxidizing conditions. Group III consisted of organisms with both high- and low-affinity cytochrome oxidases, allowing survival across a broad oxygen gradient (e.g., *Propionibacterium acnes*). Group IV represented facultative anaerobes encoding only high-affinity microaerobic cytochrome oxidases, allowing growth under low-oxygen, moderately oxidizing conditions (e.g., Lactococcus lactis, Streptococcus thermophilus). Anaerobes belonging to Group V-a (Clostridium intestinale, Akkermansia, Bacteroides, Parabacteroides, Prevotella) are devoid of both high- and low-affinity cytochrome oxidases but possess genes encoding non-heme catalase. Group V-b comprised strict anaerobes lacking both cytochrome oxidases and catalase (e.g., *Bifidobacterium, Bacteroides, Clostridium, Faecalibacterium*), indicative of a strongly reducing mucosal environment. The MRI was calculated as the ratio of (relative abundance of Group I + Group II) / (relative abundance of Group V-a + V-b). Higher MRI values, therefore, reflect a shift toward oxygen-tolerant taxa and a more oxidizing duodenal microenvironment, whereas lower values indicate anaerobic dominance and a reducing mucosal niche. Oxygen-phenotype assignments were cross-validated using literature-curated redox-associated genes, including cytochrome oxidases, superoxide dismutase, and catalase. The MRI served as a functional proxy for mucosal redox remodeling, a process hypothesized to contribute to downstream disruption of the gut–iron–immune axis in IDA.

Differentially abundant species were identified using LEfSe (LDA >3.5; microbiomeMarker package) (26, 27). Spearman correlations between CLR-transformed microbial abundances and clinical parameters (hemoglobin, leukocyte subsets, and iron indices) were computed with Hmisc (28) and visualized with corrplot (29). Microbial co-occurrence networks were inferred using SparCC. All microbiome analyses were performed in R (30).

### Transcriptomic data processing and differential expression analysis

Raw sequencing reads were quality-assessed using FastQC v0.11.9 and trimmed with Trimmomatic v0.39 (SLIDINGWINDOW:4:20, MINLEN:50) (31). High-quality reads were aligned to the human reference genome (GRCh38/hg38) using STAR v2.7.10a with GENCODE v40 annotation, and gene-level counts were quantified using featureCounts v2.0.1 (32). Low-expression genes (< 10 total counts) were excluded before downstream analysis. Differential expression analysis was performed using DESeq2 v1.48.1 with median-ratio normalization (33). In accordance with our sample-size power estimates, genes were considered significantly differentially expressed if they had a *P* < 0.05 and an absolute log_2_ fold change ≥1.5, a threshold selected to capture large-effect perturbations in iron-handling, inflammatory, and epithelial stress-response pathways. Functional enrichment analyses (Gene Ontology and KEGG) were performed using clusterProfiler, and cytokine pathway modulation was inferred using CytoSig. Spearman correlations between the top 200 microbial species and all DEGs (202 upregulated, 106 downregulated) yielded 40,400 correlations in IDA and 21,200 in controls, with 54 and 59 significant associations (*p* < 0.001), respectively. All transcriptomic analyses were performed in R.

### Integrative network analysis

To identify regulatory nodes linking the duodenal microbiome, host transcriptome, and hematological parameters, we performed an integrative correlation-based network analysis. The network incorporated three data layers: (i) relative abundances of 233 bacterial species, comprising the top 200 taxa and species contributing to beta-diversity differences; (ii) DESeq2-normalized expression values of selected host genes, including iron-metabolism genes (*TFRC, SLC40A1, BMPR2, DMT1, HAMP*), immune-related genes (*IL17A, IGHV1-45, FPR1, HLA-G*), epithelial-barrier genes (*TJP1, CDH11, CLDN5*), hypoxia-responsive genes (*EGLN3, COX7A1*), and all detected long non-coding RNAs (lncRNAs); and (iii) hematological parameters [hemoglobin, serum ferritin, transferrin saturation, and neutrophil-to-lymphocyte ratio (NLR)]. Pairwise associations were quantified using Spearman rank correlation coefficients (ρ). Correlations were considered significant if |ρ| > 0.75 (*P* < 0.001). Significant correlations were visualized in Cytoscape v3.9.1 (34), where nodes represented microbial taxa, host genes, or hematological parameters representing putative regulatory points within the gut–iron–immune interaction network.

### Statistical analysis

Statistical analyses were performed using R v4.2.2. Continuous variables were tested for normality using the Shapiro–Wilk test. Normally distributed variables were compared using two-tailed Student *t* tests and reported as mean ± SD, whereas non-normally distributed variables were compared using Mann–Whitney *U*-tests and reported as median (interquartile range). Categorical variables were compared using Fisher's exact tests. Correlations were assessed using Spearman's rank correlation coefficients Statistical significance was defined as *P* < 0.001 (two-tailed) unless otherwise specified. All microbiome and transcriptomic analyses were performed in a blinded manner, with group assignments revealed only after completion of data processing and quality control.

## Results

### Cohort characteristics

A total of 20 women with dyspepsia aged 18–40 years were enrolled and stratified into the iron-deficiency anemia (IDA) group (*n* = 11) and the control group (*n* = 9) based on WHO criteria. IDA participants exhibited marked systemic iron depletion, with significantly lower hemoglobin (10.02 ± 0.82 vs. 12.69 ± 0.67 g/dL), serum ferritin [median (IQR): 10.7 (8.2–35.3) vs. 49.7 (28.4–58.7) ng/mL], and transferrin saturation [5.52 (2.8–10.7) vs. 18.79 (15–23.8)%; all *P* < 0.05) compared with controls (Table 1). Because participants with CRP > 4 mg/L were excluded, the cohort represents a low-inflammation population. Consequently, NLR was evaluated as a continuous marker of subclinical immune tone to examine its ecological association with duodenal microbial structure, which was significantly lower in IDA subjects (1.93 ± 0.75 vs. 3.2 ± 1.8; *P* = 0.02). The groups were otherwise comparable in age (36.73 ± 8.61 vs. 34.34 ± 9.77 years; *P* = 0.60), body mass index (22.12 ± 4.73 vs. 24.71 ± 5.95 kg/m^2^; *P* = 0.36), and dietary patterns (non-vegetarian: 81% vs. 90%; *P* = 1.0), minimizing potential confounding from nutritional or metabolic factors. Comprehensive upper gastrointestinal endoscopy revealed no mucosal pathology in any participant.

**Table 1:** Baseline clinical and hematological characteristics of study participants.

Parameter	Mean ±SD/ Median (IQR) IDA	Mean ±SD/ Median (IQR) control
Age	36.73 ± 8.61	34.34 ± 9.77
BMI	24.12 ± 4.73	24.71 ± 5.95
CRP (mg/L)	1.07 (0.8–1.8)	1.64 (0.9–2.6)
Serum Iron (μg/dL)	25.6 (15.3–46.6)	74.4 (52.7–87.4)
UIBC (μg/dL)	426.9 ± 66.79	300.6 ± 25.03
TIBC (μg/dL)	455.73 ± 54.85	367.4 ± 24.79
Ferritin (ng/mL)	10.7 (8.2–35.3)	49.7 (28.4–58.7)
TSAT %	5.52 (2.8–10.7)	18.79 (15–23.8)
Hb (gm/dL)	10.02 ± 0.82	12.69 ± 0.67
Leeds dyspepsia questionnaire (LDQ)	12.78 ± 5.04	13.64 ± 8.63
Glasgow dyspepsia severity score	10.89 ± 6.11	9.55 ± 4.32

Normality was assessed using the Shapiro–Wilk test. Normally distributed variables are presented as mean ± standard deviation (SD) and compared using the unpaired *t*-test. Non-normally distributed variables are presented as medians with interquartile ranges (IQRs) and compared using the Mann–Whitney U test. Statistical significance was defined as *P* < 0.05. The *P*-values for LDQ and Glasgow dyspepsia severity scores were 0.78 and 0.90, respectively. BMI, body mass index; CRP, C-reactive protein; Hb, hemoglobin; UIBC, unsaturated iron-binding capacity; TIBC, total iron-binding capacity; TSAT, transferrin saturation.

### Duodenal dysbiosis and compositional remodelling in iron deficiency anemia

**Figure 2:**
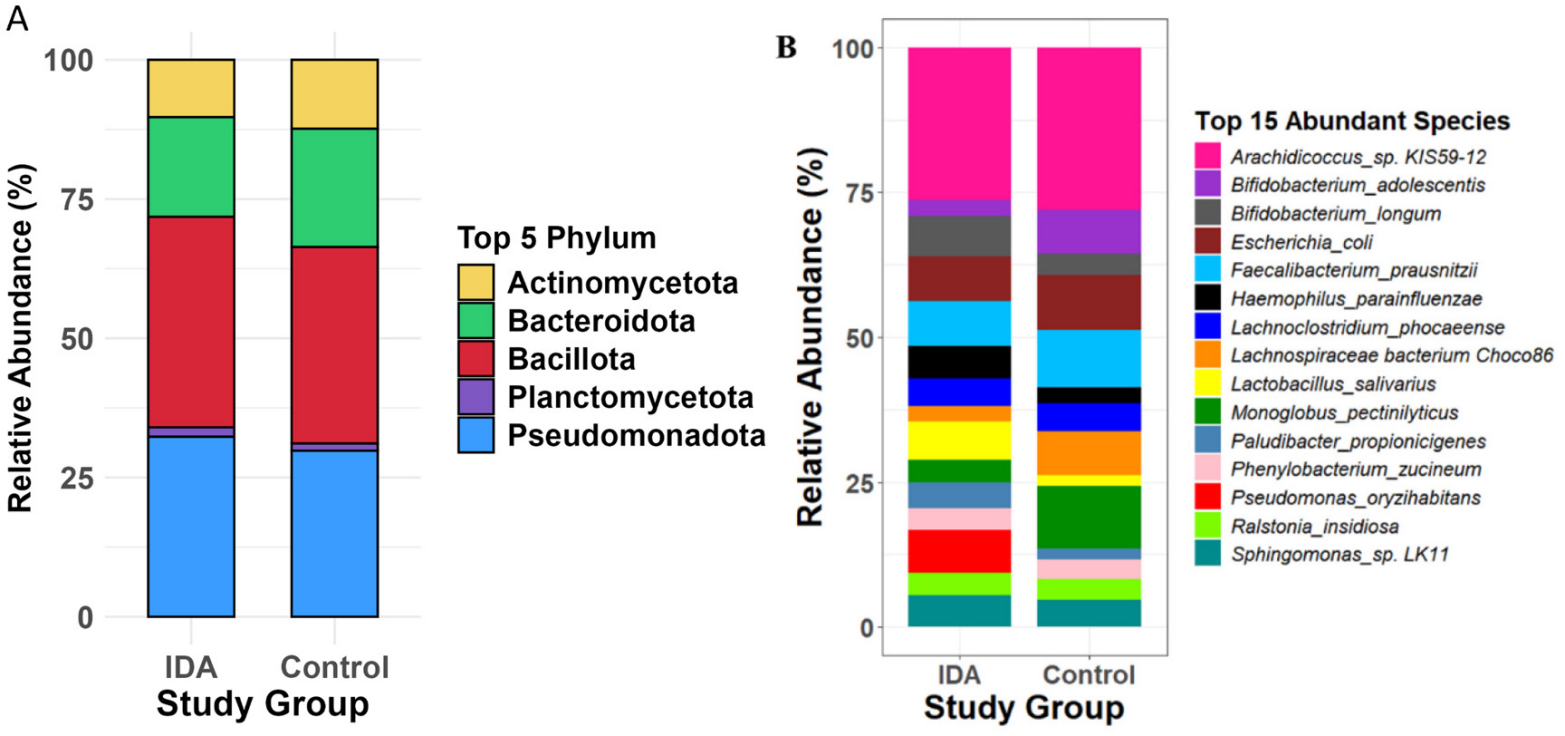
Duodenal mucosal microbiota composition in IDA and controls. **(A)** Relative abundance of major bacterial phyla. **(B)** Relative abundance of the top 15 species. IDA shows enrichment of oxygen-tolerant taxa and depletion of obligate anaerobes. Taxa are color-coded by phylum. IDA, iron deficiency anemia.

Microbial profiling of duodenal mucosal biopsies identified 2,489 operational taxonomic units (OTUs) with a mean sequencing depth of 80,541 reads per subject. At the phylum level, both groups were dominated by Bacillota, Pseudomonadota, Bacteroidota and Actinomycetota (Figure 2A). The Pseudomonadota: Bacteroidota ratio was higher in IDA (0.53 ± 0.38 vs 0.31 ± 0.17), although this difference did not reach statistical significance (*P* = 0.21; Supplementary Figure 1).

At the species level, IDA subjects exhibited distinct compositional remodeling (Figure 2B; Supplementary Figure 2). The oxygen-tolerant *Lactobacillus salivarius* was markedly enriched in IDA (6.77% vs. 1.81%), whereas several beneficial obligate anaerobes were depleted, including *Bifidobacterium adolescentis* (2.86% vs. 7.56%) and *Faecalibacterium prausnitzii* (7.78% vs. 9.94%). Notably, while *Bifidobacterium longum* was increased in IDA (6.86% vs. 3.69%), the loss of other key anaerobes, such as *Monoglobus pectinolyticus*, contributed to altered community composition. Collectively, these shifts shows restructuring of the duodenal microbial community in IDA, with expansion of oxygen-tolerant taxa and loss of several core anaerobes.

**Figure 3:**
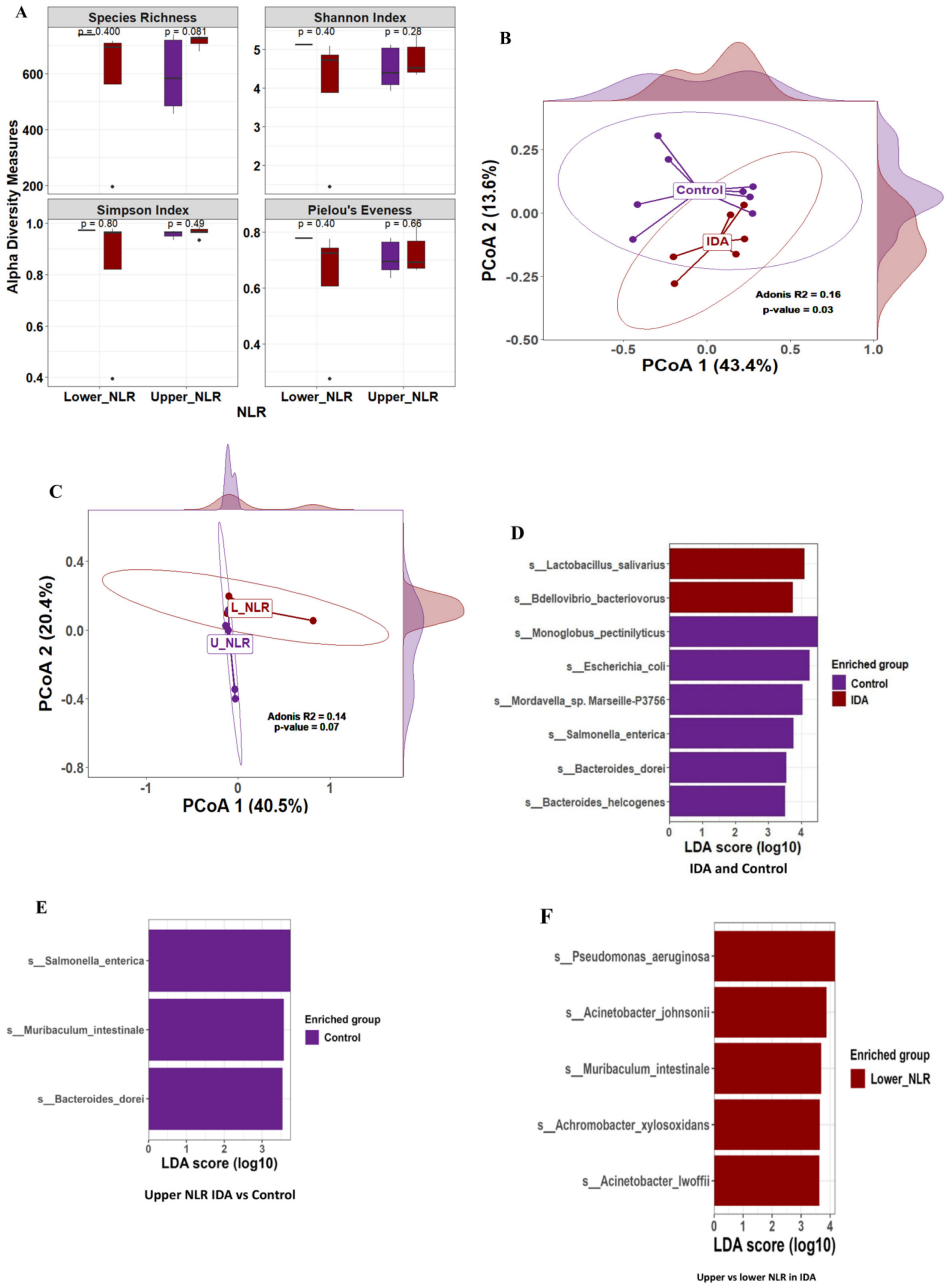
Microbial diversity and discriminative taxa stratified by NLR status. **(A)** α-diversity metrics (Observed richness, Shannon, Simpson, Pielou's evenness) comparing IDA and controls within high- and low-NLR strata. **(B)** β-diversity (Bray–Curtis PCoA) comparing high-NLR IDA and controls. **(C)** β-diversity comparing high- vs. low-NLR subgroups within IDA. **(D–F)** LEfSe analysis identifying discriminative taxa between **(D)** IDA vs. controls, **(E)** high-NLR IDA vs. controls, and **(F)** high- vs. low-NLR IDA. LDA scores > 3.5 indicate significantly enriched taxa.

To examine how subclinical immune status relates to duodenal microbial ecology, participants were stratified by NLR status. Within the high-NLR subset, IDA subjects showed a trend toward greater species richness (716 ± 22 vs. 596 ± 125 OTUs; *P* = 0.08; Figure 3A), while other α-diversity metrics (Shannon, Simpson, Pielou's evenness) remained comparable.

β diversity analysis using Bray–Curtis dissimilarity and PCoA revealed distinct microbial community structures between IDA and controls within the high-NLR stratum (Bray–Curtis; PERMANOVA *P* = 0.03, R^2^ = 0.16; Figure 3B). Within the IDA cohort, microbial communities were further segregated by NLR status (PERMANOVA *P* = 0.07, R^2^ = 0.14; Figure 3C), indicating that microbial composition is linked to variations in immune tone, even in the absence of systemic inflammation. Species contributing to β-diversity differences are listed in Supplementary Table 1.

### Loss of mucosal anaerobiosis and microbial redox remodelling

LEfSe analysis identified 16 discriminative taxa (LDA > 3.5) that robustly distinguish the IDA duodenal mucosa from controls (Figures 3D–F). IDA subjects were characterized by a significant depletion of several obligate anaerobes and key commensals, including *Bacteroides dorei* (LDA = 3.4, *P* = 0.017), *Monoglobus pectinilyticus* (LDA = 4.5, *P* = 0.04), *Escherichia coli (*LDA = 4.2, *P* = 0.02)*, Mordavella sp. Marseille-P3756* (LDA = 4.02, *P* = 0.03)*, and Bacteroides helcogenes* (LDA = 3.16, *P* = 0.01), all of which were enriched in controls. Conversely, the IDA niche was dominated by oxygen-tolerant and microaerophilic taxa, most notably *Lactobacillus salivarius* (LDA = 4.1, *P* = 0.02), Bdellovibrio *bacteriovorus* (LDA = 3.7, *P* = 0.04).

This pattern reflects loss of obligate anaerobes alongside expansion of aerobic and siderophore-producing taxa. These taxonomic shifts are consistent with altered microbial community structure in IDA.

To quantify shifts in microbial respiratory phenotypes, we calculated the Microbial Redox Index (MRI), defined as the ratio of oxygen-tolerant taxa (Groups I + II) to anaerobic taxa (Groups V-a + V-b). The overall duodenal MRI did not differ significantly between groups, with median values of 2.79 (1.58–6.07) in IDA subjects vs. 3.95 (0.66–4.80) in controls (*P* = 0.44; Supplementary Figure 4A).

**Figure 4:**
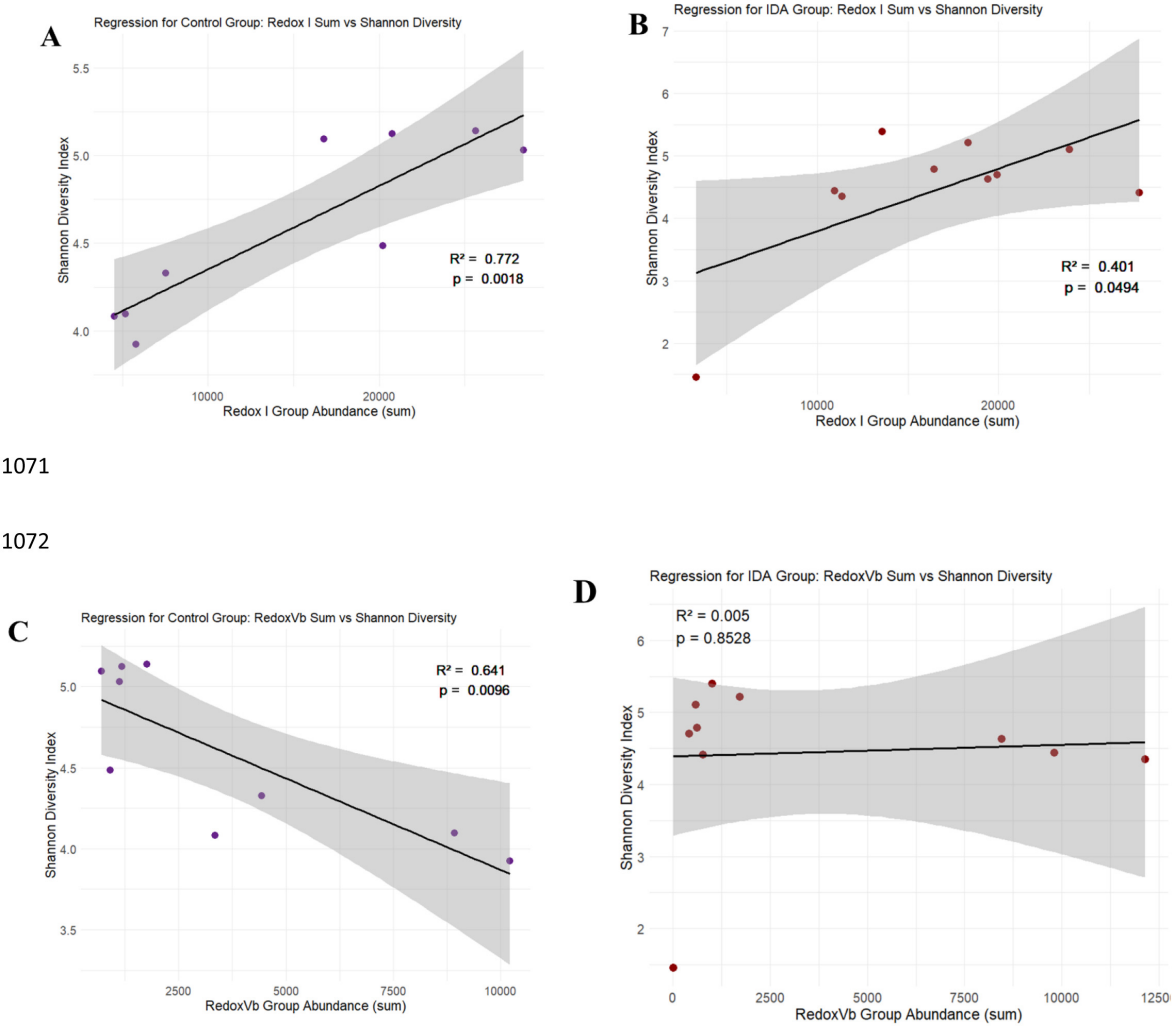
Associations between microbial redox phenotype, diversity, and host gene expression. **(A–D)** Linear regression of Shannon diversity against redox phenotype groups: **(A)** Group I in IDA, **(B)** Group I in controls, **(C)** Group Vb in controls, and **(D)** Group Vb in IDA.

Despite these similar overall ratios (Figures 4A, B), regression analyses revealed altered associations between microbial groups and diversity metrics. In healthy controls, Redox Group V-b anaerobes exhibited a strong inverse relationship with Shannon diversity (R^2^ = 0.64, *P* = 0.009; Figure 4C).

In contrast, this correlation was not observed in IDA cohort, where the correlation was lost (Figure 4D; Supplementary Figures 4B, C). Conversely, Redox Group I aerobes showed a consistent positive correlation with Shannon diversity across both groups (R^2^ = 0.77, *P* = 0.001; Figure 4A), (R^2^ = 0.40, *p* = 0.04; Figure 4B).

The regression analysis with Observed OTUs mirrored the patterns seen with the Shannon index: Redox Group I was positively associated with species richness in both Control (R^2^ = 0.912, *p* = 1e−04) and IDA groups (R^2^ = 0.529, *p* = 0.017) Conversely, Redox Group Vb showed a negative association in the Control group (R^2^ = 0.70, *p* = 0.0049) that was notably lost in IDA group (R^2^ = 0.059, *p* = 0.499) (Supplementary Figures 4D–G).

### Transcriptomic profiling reveals Th17-driven immune reprogramming and epithelial barrier dysfunction

**Figure 5:**
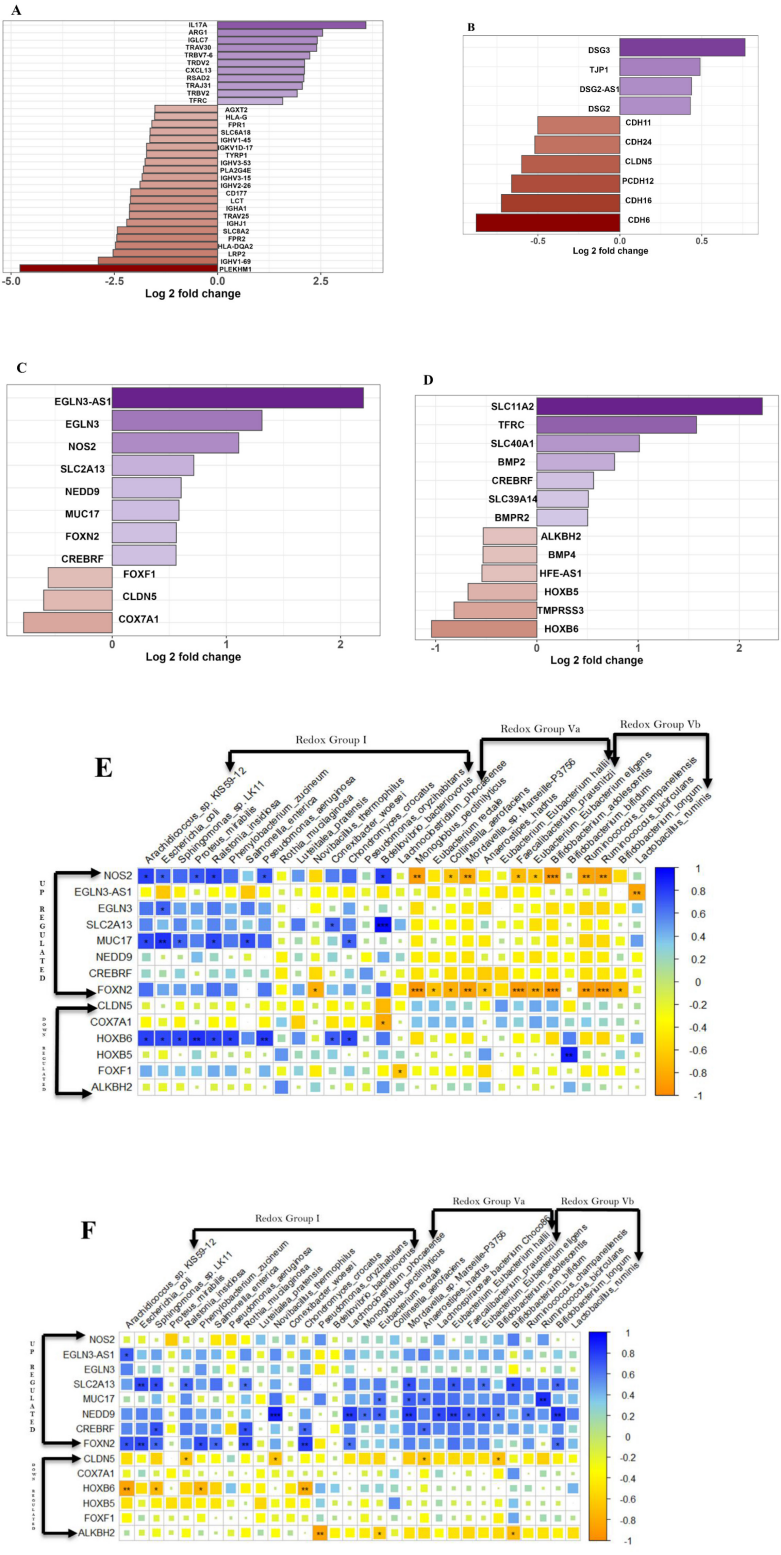
Differential gene expression in duodenal mucosa of IDA subjects. Bar plots showing log_2_ fold-change in genes related to **(A)** immune signaling, **(B)** tight-junction and barrier integrity, **(C)** hypoxia response, and **(D)** iron metabolism. Blue bars denote upregulated genes; red bars denote downregulated genes. **(E, F)** Heatmaps showing Spearman correlations between redox groups and host genes in **(E)** controls and **(F)** IDA. Color scale reflects correlation coefficients (−1 to +1). *P* < 0.05 (*), P* < 0.01 (*), P < 0.001 (**).

RNA-Seq of duodenal biopsies identified 5,094 differentially expressed genes (DEGs) between IDA and controls (*p* < 0.05, |log_2_FC| > 1; Supplementary Figures 3A–E). Gene Ontology enrichment demonstrated extensive remodeling of immune response pathways (FDR = 2.3 × 10^−15^; Figure 5A), epithelial barrier function (FDR = 1.8 × 10^−15^; Figure 5B), and metabolic processes (FDR = 4.5 × 10^−10^; Figure 5C), showing widespread differences in gene expression between groups.

The IDA mucosa exhibited a pronounced Th17-polarized inflammatory signature, with significant upregulation of IL17A (log_2_FC = +3.59, *P* < 0.001), TRAV30 (log_2_FC = +2.4, *P* < 0.01), and TRDV2 (log_2_FC = +2.1, *P* = 0.0009), reflecting activation of Th17 and γδ T-cell pathways (Figure 5A). Conversely, markers of adaptive humoral defense and immunoglobulin assembly were suppressed, including IGHV1-45 (log_2_FC = −1.64, *P* < 0.02), FPR1 (log_2_FC = −1.59, *P* = 0.009), and HLA-G (log_2_FC = −1.52, *P* = 0.01).

This pattern reflects recurrent upregulation of Th17 related genes and downregulation of IgA/IgG-associated transcripts.

### Hypoxic stress and the iron-trapping phenotype

Evidence of epithelial barrier disruption was prominent (Figure 5B). While TJP1 (ZO-1) and desmosomal genes (DSG2, DSG3) were upregulated, key structural components, including CDH11, CDH6, and CLDN5 (log_2_FC = −0.59, *P* = 0.02), were significantly downregulated (35).

The IDA mucosa also demonstrated a hypoxic stress response (Figure 5C), with upregulation of EGLN3 (PHD3) (log_2_FC = +1.31, *P* < 0.0001), COX7A1, and SLC2A13. Notably, EGLN3 induction during localized inflammation can paradoxically stabilize HIF-1α, creating a molecular environment conducive to iron sequestration. KEGG analysis indicated enrichment of hypoxia-related pathways, including HIF-1 signalling, in IDA patients (Supplementary Figure 3F).

Transcriptomic profiling revealed divergent changes in mucosal iron handling pathways (Figure 5D). The IDA mucosa exhibited marked upregulation of iron-import transcripts, including SLC11A2 (DMT1) and TFRC (transferrin receptor; log_2_FC = +1.58, *P* < 0.001), a canonical signal of cellular iron starvation. Paradoxically, this was accompanied by a significant increase in SLC40A1 (ferroportin) mRNA (log_2_FC = +1.02, *P* = 0.0002) and BMPR2 (log_2_FC = +0.50, *P* = 0.0002).

### Integrated redox–transcriptome profiling reveals microbial hub taxa and a central lncRNA driver in IDA

To determine whether microbial respiratory phenotypes contribute to mucosal transcriptional remodeling, we performed Spearman correlation analysis between microbial redox groups and host differentially expressed genes (DEGs). In controls, aerobic taxa (Group I) positively correlated with immune regulatory genes, whereas anaerobic taxa (Groups Va/Vb) were negatively associated with stress- and inflammation-linked transcripts, reflecting a balanced, homeostatic microbe–host interface (Figure 5E).

In IDA, these relationships were disrupted. Both aerobic and anaerobic taxa displayed positive correlations with transcripts associated with Th17-skewed inflammation (IL17A), hypoxic stress (EGLN3), and sensitized BMP signaling (BMPR2). Conversely, these taxa correlated negatively with downregulated immune-regulatory genes (Figure 5F).

To identify microbial taxa associated with host transcriptional changes, we constructed an integrative correlation network for the IDA cohort, incorporating 38 host genes significantly associated with microbial taxa, compared with only 13 in the control network (Supplementary Figures 5A–D). Within this expanded IDA network, three oxygen-tolerant bacteria emerged as highly connected hub taxa (≥7 edges).

**Figure 6:**
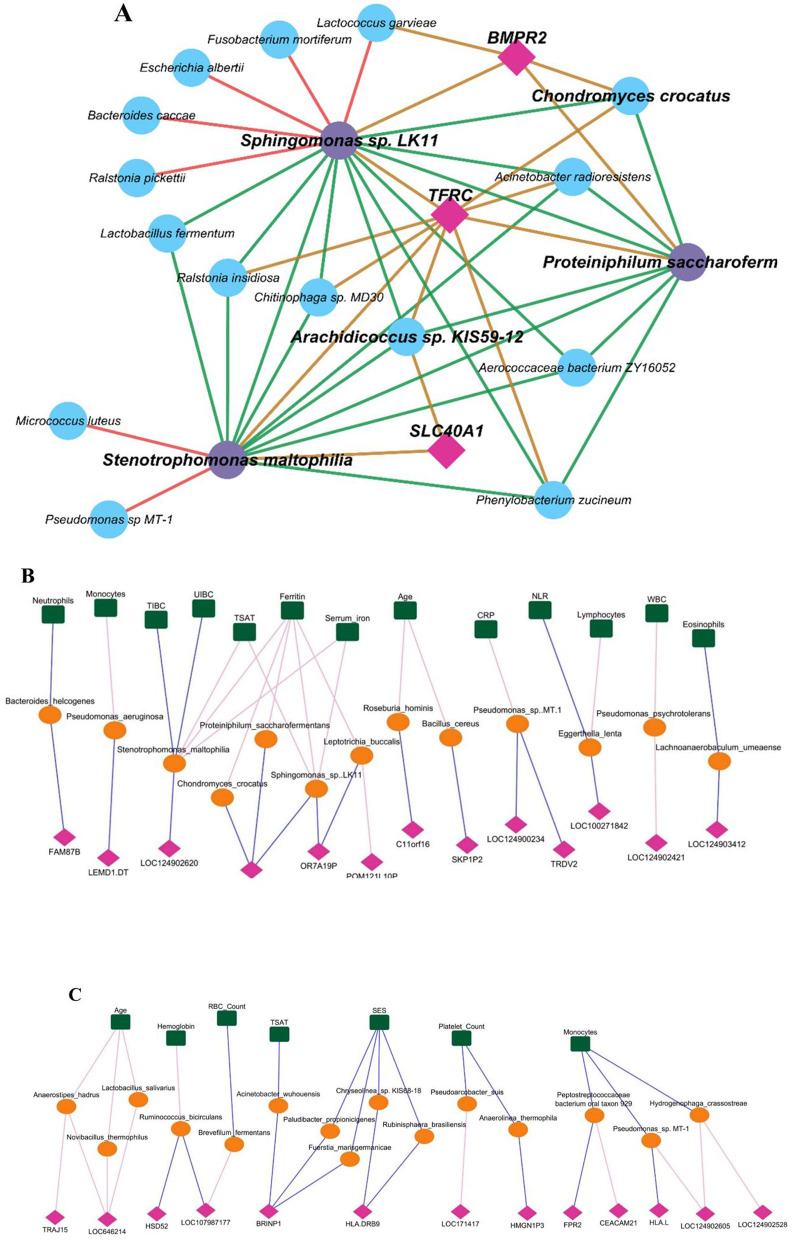
Integrated host–microbe–hematological interaction networks. **(A)** Microbial–microbial and microbe–gene associations. Pink diamonds represent host genes; purple circles denote microbial hub taxa; blue circles represent other taxa. Brown edges indicate gene–microbe correlations; green edges denote multi-edge microbe–microbe interactions; red edges denote single interactions. **(B, C)** Correlation networks integrating hematological parameters (green squares), microbial taxa (orange circles), and host genes (pink diamonds) in **(B)** IDA and **(C)** controls. Blue edges indicate positive correlations; pink edges indicate negative correlations.

*Sphingomonas sp. LK11* (21 connections) showed strong associations with iron-regulatory pathways, microbes, and systemic iron status, correlating positively with TFRC (ρ = 0.78, *P* = 0.007), BMPR2 (ρ = 0.67, *P* = 0.03), and with SLC40A1 (ρ = 0.70, *P* = 0.02) via *Arachidicoccus sp. KIS59-12* and inversely with serum iron (ρ = −0.82, *P* = 0.003), ferritin (ρ = −0.79, *P* = 0.006), and TSAT (ρ = −0.79, *P* = 0.006). *Stenotrophomonas maltophilia* (18 connections) was correlating with SLC40A1 (ρ = 0.70, *P* = 0.02) and BMPR2 (ρ = 0.6, *P* = 0.06), inversely with ferritin (ρ = −0.86, *P* = 0.001), serum iron (ρ = −0.79, *P* = 0.006), TSAT (ρ = −0.84, *P* = 0.002) and positively with TIBC (ρ = 0.79, *P* = 0.006) and UIBC (ρ = 0.78, *P* = 0.008). *Proteiniphilum saccharofermentans* (10 connections) correlates positively with TFRC (ρ = 0.79, *P* = 0.006), BMPR2 (ρ = 0.66, *P* = 0.03), and negatively with Ferritin (ρ = −0.79, *P* = 0.006) (Figures 6A,B).

The most prominent feature of the IDA network was the emergence of LOC124902620, a previously uncharacterized long non-coding RNA (Figure 6B). Its expression correlated with *Stenotrophomonas maltophilia* (ρ = 0.62, *P* = 0.05), which showed strong inverse correlations with Ferritin (ρ = −0.86, *P* = 0.001), serum iron (ρ = −0.79, *P* = 0.006), and transferrin saturation (ρ = −0.84, *P* = 0.002). These multilayered interactions were absent in the control network (Figure 6C), where LOC124902620 remained a peripheral, weakly connected node with minimal links to microbial taxa or iron-related genes.

## Discussion

This multi-omic investigation positions iron deficiency anemia (IDA) as a disorder rooted in duodenal mucosal dysfunction rather than a simple systemic iron deficiency. By integrating microbial, transcriptional, and epithelial signatures, our findings suggest coordinated mucosal program in which dysbiosis-driven immune polarization, microbial redox remodeling, and localized hepcidin-mediated ferroportin degradation converge to disrupt epithelial iron export. Together, these processes are consistent with a possible enterocyte iron retention phenotype, a paradoxical state in which enterocytes exhibit intracellular iron scarcity while simultaneously failing to release absorbed dietary iron into the circulation. This integrated framework provides a biologically coherent explanation for the 30–50% inadequate response to the oral iron therapy observed in IDA patients (36).

The bidirectional disruption of enterocyte iron trafficking, marked by transferrin receptor upregulation alongside transcriptomic signatures consistent with a functional blockade of basolateral iron export, emerges as a proposed central pathogenic mechanism in IDA. Transcriptional profiling in this cohort reveals a coherent “starvation-amidst-blockade” phenotype that redefines mucosal iron handling. Transferrin receptor (TFRC/CD71) (37) Expression is post-transcriptionally regulated by iron regulatory proteins (IRP1/IRP2) (38), which stabilize TFRC mRNA under low intracellular iron conditions, thereby enhancing basolateral iron uptake from circulating transferrin. The marked TFRC upregulation (log_2_FC = +1.58) indicates that enterocytes are functionally iron-starved, effectively increasing their demand for iron from the systemic circulation despite ongoing luminal absorption.

Paradoxically, SLC40A1 (ferroportin) mRNA is also significantly upregulated (log_2_FC = +1.02, *P* = 0.0002), likely reflecting a compensatory attempt to enhance basolateral iron export. However, this transcriptional response occurs within a microenvironment characterised by markedly elevated IL17A (log_2_FC = +3.59), EGLN3 (log_2_FC = +1.31), and BMPR2, three pathways known to converge on post-translational ferroportin degradation. As a result, despite increased mRNA, ferroportin protein is unlikely to accumulate at the cell surface. In this inflammatory and hypoxic microenvironment, ferroportin is expected to undergo hepcidin-mediated internalization, cytokine-driven ubiquitination, and BMP–SMAD–dependent suppression, (39, 40) resulting in a functional loss of basolateral export capacity. Meanwhile, apical iron uptake remains intact, as evidenced by significant upregulation of SLC11A2/DMT1 (log_2_FC = +2.2). With import preserved and export impaired, dietary iron accumulates intracellularly, where it undergoes Fenton-driven redox cycling, generating reactive oxygen species. This proposed oxidative stress could exacerbate epithelial barrier dysfunction and mucosal inflammation, further inducing hepcidin and intensifying ferroportin degradation in a self-perpetuating cycle. Together, these processes drive intracellular iron sequestration, a mucosal iron-retention state that explains both the high failure rate of oral iron therapy and the gastrointestinal side effects experienced by patients, as iron-induced oxidative injury occurs within an already-stressed duodenal epithelium.

A central question arising from this study is whether duodenal dysbiosis is a primary cause or a secondary consequence of iron-deficiency anemia (IDA). Our findings support a bidirectional, feed-forward relationship in which dysbiosis and impaired mucosal iron handling reinforce one another. On one hand, dysbiosis appears to drive IDA actively: the Microbial Redox Index (MRI) shows a shift toward a more oxidizing mucosal environment, accompanied by the enrichment of oxygen-tolerant, siderophore-producing taxa such as *Sphingomonas* and *Stenotrophomonas*. These pathobionts correlate strongly with IL17A, EGLN3, and BMPR2, linking them to inflammatory and hypoxia-responsive (39) pathways that promote hepcidin induction and ferroportin degradation. Functionally, these organisms can reduce luminal iron bioavailability by oxidizing Fe^2+^ to Fe^2+^ and by directly competing with the host for iron via siderophores, thereby amplifying epithelial iron starvation. Conversely, the iron-deficient mucosal environment itself fosters dysbiosis: suppressed adaptive immunity (e.g., downregulation of IGHV1-45), barrier dysfunction, and a pro-inflammatory niche that selectively favors oxygen-tolerant opportunists and iron-scavenging bacteria, including *Lactobacillus* and *Bifidobacterium* that thrive under conditions of reduced systemic iron availability. Together, these observations suggest a possible self-reinforcing pathological loop in which an initial dysbiotic insult triggered by diet, antibiotics, or infection impairs iron absorption and induces localized inflammation; systemic iron deficiency then develops; and the resulting immune dysfunction and barrier breach further worsen dysbiosis, which, in turn could intensify the mucosal iron sequestration phenotype. This framework explains why a subset of IDA patients fails to respond to oral iron supplementation alone: in such cases, correction of dysbiosis and mucosal inflammation is essential to restore effective basolateral iron export.

The Microbial Redox Index (MRI) emerged as one of the most informative microbiome-derived signals in this study. Although the overall ratio of oxygen-tolerant to anaerobic taxa appeared comparable between groups at the broad community level (*P* = 0.44), regression and subgroup analyses revealed that MRI is a sensitive functional readout of duodenal redox remodeling. A shift toward a more oxidizing mucosal niche has several important implications for iron handling. Oxygen-tolerant organisms can generate reactive oxygen species that oxidize ferrous iron (Fe^2+^) to ferric iron (Fe^2+^), thereby reducing its bioavailability, as DMT1 preferentially transports the ferrous form. Many of the aerobic and oxygen-tolerant taxa enriched in IDA, most notably *Stenotrophomonas*, produce high-affinity siderophores that directly compete with the host for luminal iron. In parallel, the depletion of butyrate-producing obligate anaerobes, such as Faecalibacterium prausnitzii, compromises epithelial barrier integrity, amplifying localized inflammation and promoting hepcidin-mediated degradation of ferroportin (41). Together, these features position the MRI as a functional biomarker of dysbiosis-associated redox stress and a promising precision tool for stratifying IDA phenotypes. A high MRI may indicate a dysbiosis- and inflammation-driven form of IDA that is likely refractory to standard oral iron and may require microbiome-targeted therapy. In contrast, a low MRI may reflect a predominantly nutritional deficiency that responds to supplementation alone. This framework provides a path toward personalized clinical management for the nearly 2 billion individuals worldwide affected by IDA.

The identification of LOC124902620 as the most highly connected node in the integrative network (Figure 6B) represents a significant advance in understanding the mucosal response to iron deficiency. This previously uncharacterized lncRNA showed strong inverse correlations with systemic iron indices, including Ferritin (ρ = −0.86), serum iron (ρ = −0.79), and transferrin saturation (ρ = −0.84) in Stenotrophomonas, indicating that its expression is directly associated with the clinical severity of iron depletion. Its levels were also tightly linked to dysbiotic taxa, displaying positive associations with *Stenotrophomonas maltophilia* and a negative association with the beneficial commensal *Bifidobacterium bifidum*. Importantly, LOC124902620 correlated with key epithelial iron-handling genes, including TFRC and SLC40A1, positioning it at the intersection of microbial, immune, and epithelial iron-export pathways. Together, these multilayered associations suggest that LOC124902620 functions as a microbial-responsive regulatory element, a molecular bridge integrating dysbiosis-derived signals with the epithelial machinery that maintains the enterocyte iron retention. If future CRISPR-based perturbation or *in vivo* knockdown studies confirm a causal role, this lncRNA may emerge as a promising therapeutic target for restoring ferroportin-mediated export in refractory IDA.

The emergence of *Sphingomonas* sp., *Stenotrophomonas maltophilia*, and *Proteiniphilum saccharofermentans* as central hub taxa highlights their role as active orchestrators of the dysbiosis–iron axis. All three oxygen-tolerant organisms were significantly enriched in the IDA mucosa, and their high network connectivity suggests coordinated interference with pathways governing iron uptake, export, and localized inflammation. *Sphingomonas* showed strong associations with SLC40A1 and TFRC, while *Stenotrophomonas maltophilia* correlated tightly with IL17A and other inflammatory mediators, linking microbial expansion to mucosal immune activation. The proliferation of these hub taxa likely creates a competitive luminal environment in which microbial iron acquisition is prioritized over host absorption. Mechanistically, this may occur through high-affinity siderophore production that sequesters (42) luminal iron and generates localized reactive oxygen species (ROS) (43), exacerbating epithelial stress and contributing to the hepcidin-rich microenvironment that drives ferroportin loss. A possible mechanistic model suggests these interactions reinforce the enterocyte iron sequestration and underscore the central role of microbial ecology in determining iron-absorption efficiency. Collectively, these findings suggest that reversing iron retention phenotype may require microbiome-directed interventions, such as restoring obligate anaerobes or modulating siderophore activity, alongside standard supplementation to re-establish mucosal iron export.

### Clinical implications and future directions

Our findings provide a mechanistic rationale for a multi-target therapeutic strategy aimed at re-establishing mucosal iron export. Restoring systemic iron levels in refractory IDA will likely require shifting the duodenal mucosa from a pro-inflammatory, oxidizing niche toward a homeostatic, anaerobic environment that supports efficient ferroportin-mediated export.

Ecological Restoration to Reduce the MRI: Re-establishing beneficial obligate anaerobes represents a promising avenue to reverse duodenal redox remodeling and competitively exclude oxygen-tolerant pathobionts. Multi-strain formulations containing *Bifidobacterium adolescentis* and *Bacteroides dorei* may help restore a reducing luminal environment while providing butyrate to support epithelial repair. Prior evidence that Bifidobacterium supplementation improves hemoglobin levels in anemic children underscores the translational potential of this ecological strategy approach.

Epithelial Barrier Repair: Interrupting the inflammation–hepcidin–ferroportin loop requires strengthening mucosal barrier integrity. Prebiotics, such as inulin (44) and fructooligosaccharides, direct butyrate supplementation (45), and zinc, each known to enhance tight junction stability, may reduce microbial antigen leakage, dampen mucosal inflammation, and facilitate the restoration of effective iron export.

Targeted Modulation of the Hepcidin–Ferroportin Axis: Rescuing basolateral iron efflux remains the most direct strategy for treating refractory IDA. Anti-inflammatory adjuncts, such as omega-3 fatty acids (to attenuate IL17A-driven signaling) or vitamin D (to modulate Th17 differentiation), may help rebalance mucosal immune tone. In parallel, emerging hepcidin-neutralizing antibodies and BMP-pathway inhibitors, currently in trials for other inflammatory anemias, may hold therapeutic promise for patients exhibiting a mucosal iron export blockade.

Proposed Clinical Trial Pathway: Guided by our multi-omic network, we propose a Phase II randomized controlled trial evaluating a combination restoration therapy (multi-strain probiotic + prebiotic + oral iron) versus standard iron monotherapy in women with IDA, stratified by elevated NLR. Primary endpoints would include hemoglobin normalization and a reduction in the Microbial Redox Index (MRI) at 12 weeks. If effective, this precision-medicine framework could reshape the therapeutic landscape for the nearly two billion individuals worldwide affected by IDA.

### Study limitations

Several limitations of this investigation warrant consideration. Although the sample size (11 IDA and 9 controls) was sufficient to resolve large-effect transcriptomic and microbial shifts, the small cohort limits broad generalizability, underscoring the need for validation in larger, multi-center studies across diverse geographic and ethnic populations. The cross-sectional design also precludes definitive causal inference, highlighting the importance of longitudinal studies that track microbiome and transcriptome dynamics throughout the onset, progression, and clinical resolution of IDA. In addition, while transcriptomic signatures strongly suggest a hepcidin-mediated export blockade, serum hepcidin levels were not directly measured, and duodenal iron content was not assessed histologically; future work should incorporate hepcidin ELISA and iron-staining methods, such as Perls' Prussian blue, to confirm these mechanistic inferences. Functional validation of the novel lncRNA LOC124902620 and the identified microbial hub taxa is also lacking; mechanistic studies using patient-derived duodenal organoids and gnotobiotic mouse models will be essential to establish causality within the gut–iron–immune axis. Furthermore, the cohort consisted exclusively of young women, limiting generalizability to men, children, older adults, and pregnant individuals. Finally, although dietary patterns were recorded, micronutrient intake was not comprehensively quantified; future studies should incorporate more detailed dietary surveys and food-frequency questionnaires to better disentangle nutritional factors from mucosal drivers of IDA.

## Conclusion

This multi-omic investigation reframes iron deficiency anemia as a duodenal mucosal disorder characterized by dysbiosis-driven immune polarization, microbial redox remodeling, and hepcidin-mediated ferroportin blockade. Together, these processes define the molecular architecture of the “Enterocyte Iron Trap,” a paradoxical state in which enterocytes are simultaneously starved for iron, reflected by TFRC and SLC11A2 upregulation, yet unable to export absorbed dietary iron due to ferroportin loss.

The identification of LOC124902620 as a potential regulatory node linking dysbiosis to epithelial iron handling, along with the discovery of oxygen-tolerant microbial hub taxa (Sphingomonas, Stenotrophomonas, and Proteiniphilum), highlights actionable therapeutic targets for the substantial proportion of patients who fail oral iron therapy. By “reversing the enterocyte iron trap” through precision microbiome-based, ecological, and anti-inflammatory interventions, it may be possible to transform the treatment landscape for the nearly 2 billion individuals worldwide affected by IDA.

## Data Availability

All sequencing data are publicly available and deposited under BioProject ID 710 PRJNA106257.
