# Supplementary material for: Duodenal dysbiosis is linked to altered ferroportin related transcriptomics programs in iron deficiency anemia
Source: Front Nutr. 2026 Jul 1;13:1836940. doi: 10.3389/fnut.2026.1836940 (PMC13368980; doi:10.3389/fnut.2026.1836940)
Supplement: Supplementary file 1 [file Data_Sheet_1.pdf]

Supplementary

SUPPLEMENTARY FIGURE 1

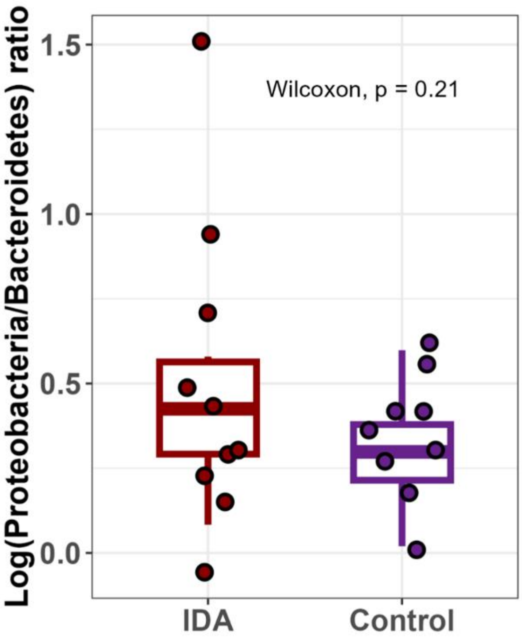

SUPPLEMENTARY FIGURE 2

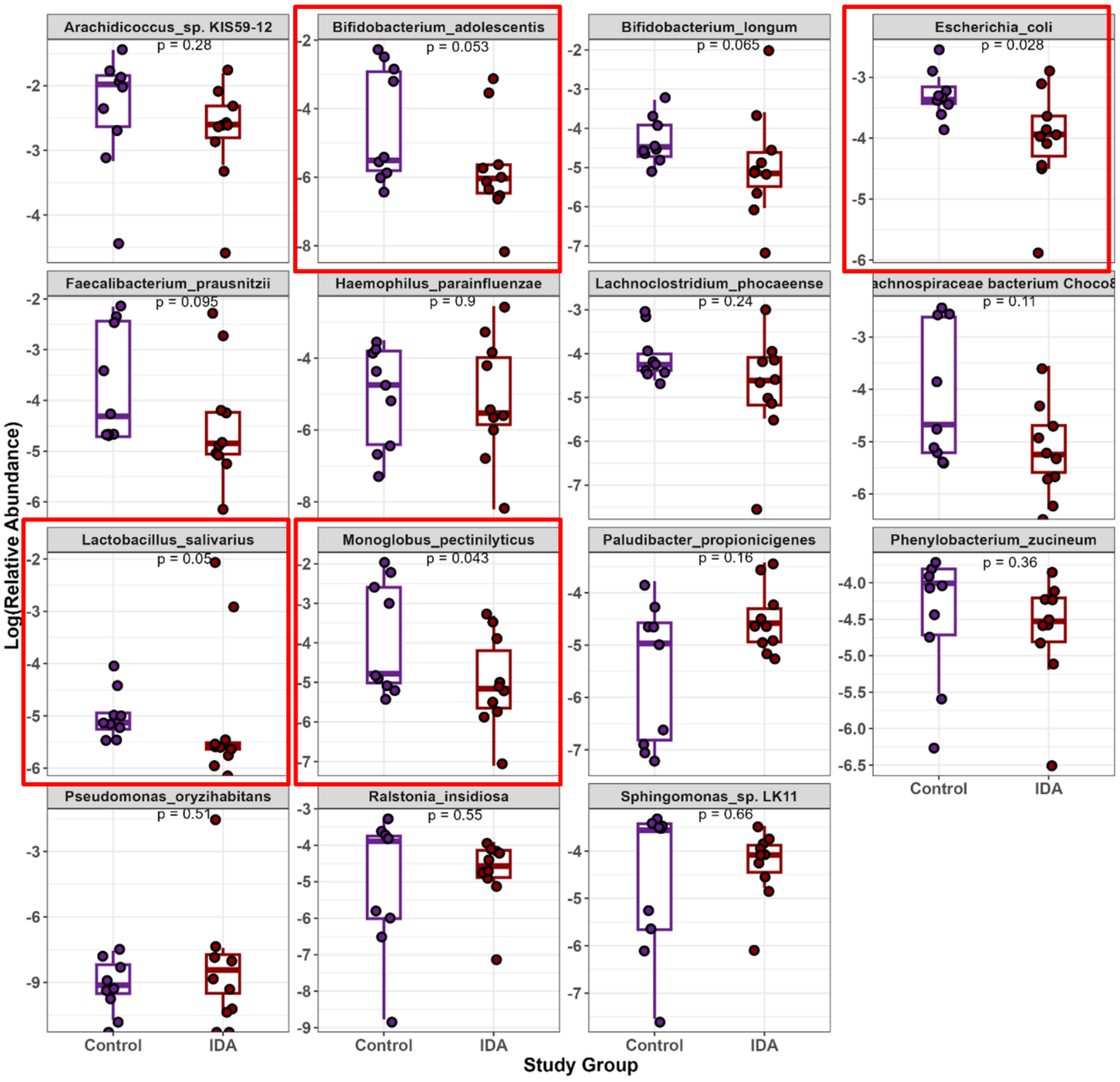

SUPPLEMENTARY FIGURE 3

A

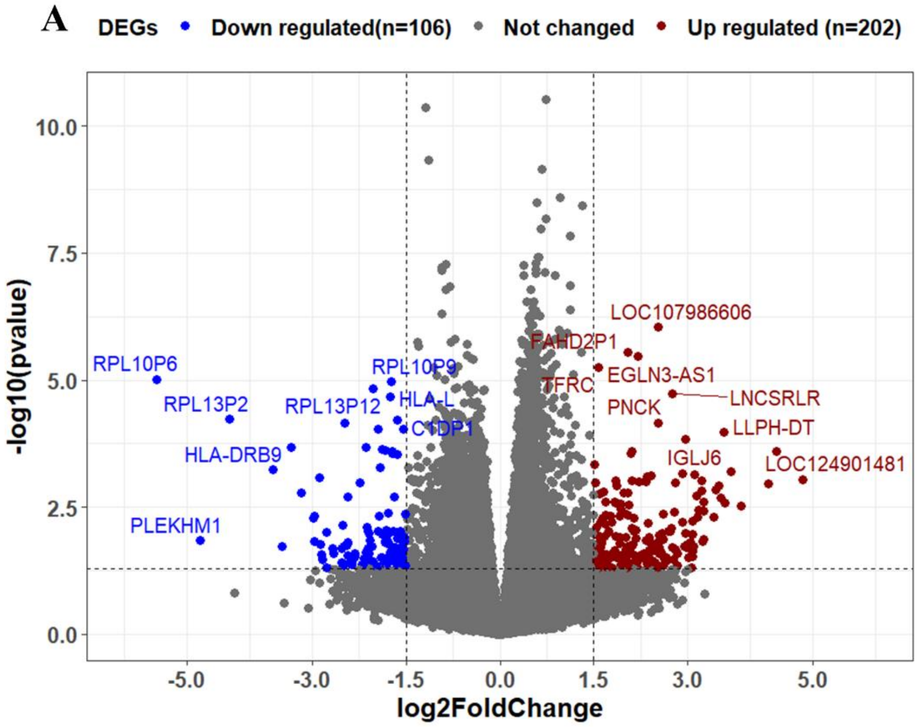

B

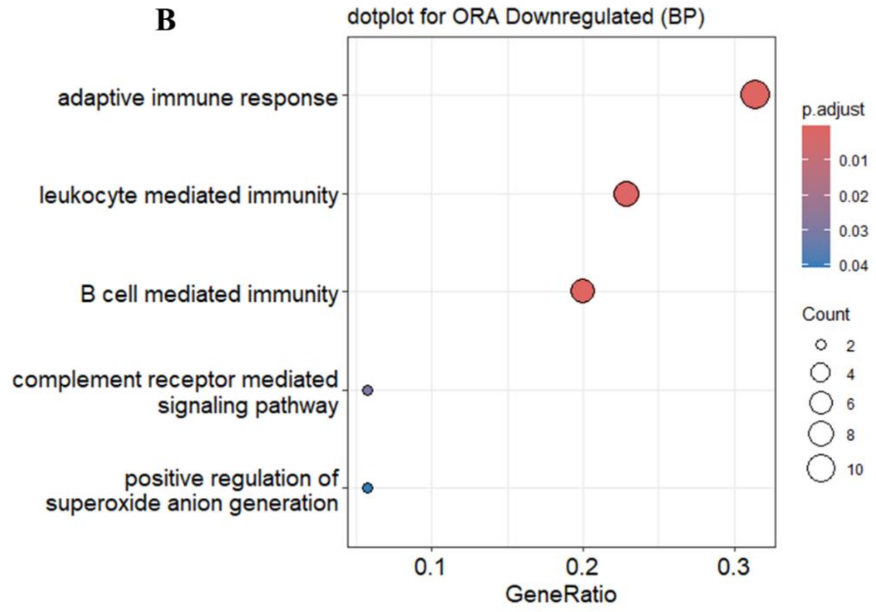

C

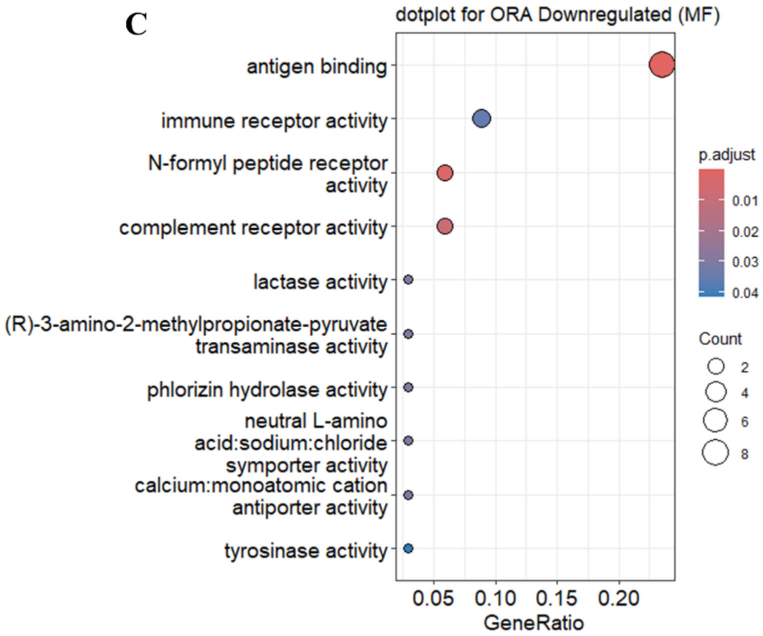
dotplot for ORA Downregulated (MF)
antigen binding
immune receptor activity
N-formyl peptide receptor activity
complement receptor activity
lactase activity
(R)-3-amino-2-methylpropionate-pyruvate transaminase activity
phlorizin hydrolase activity
neutral L-amino acid:sodium:chloride symporter activity
calcium:monoatomic cation antiporter activity
tyrosinase activity
0.05
0.10
0.15
0.20
GeneRatio
p.adjust
0.01
0.02
0.03
0.04
Count
2
4
6
8

D

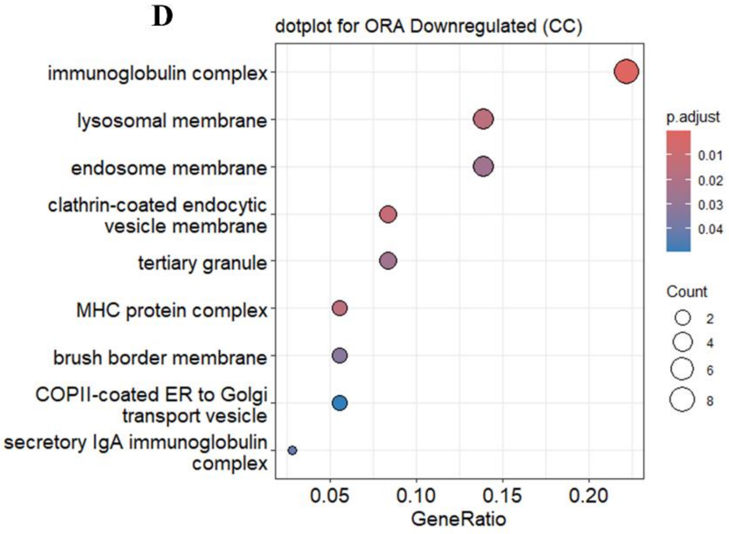

dotplot for ORA Downregulated (CC)
immunoglobulin complex
lysosomal membrane
endosome membrane
clathrin-coated endocytic vesicle membrane
tertiary granule
MHC protein complex
brush border membrane
COPII-coated ER to Golgi transport vesicle
secretory IgA immunoglobulin complex
0.05
0.10
0.15
0.20
GeneRatio
p.adjust
0.01
0.02
0.03
0.04
Count
2
4
6
8

E

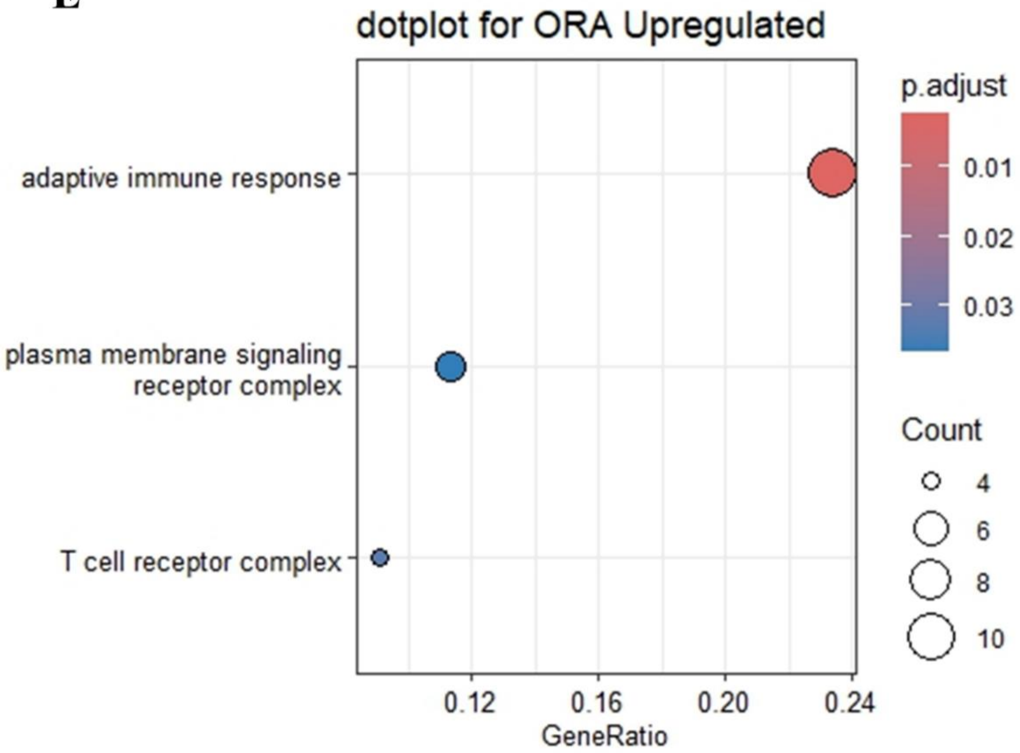

dotplot for ORA Upregulated
adaptive immune response
plasma membrane signaling receptor complex
T cell receptor complex
0.12
0.16
0.20
0.24
GeneRatio
p.adjust
0.01
0.02
0.03
Count
4
6
8
10

F

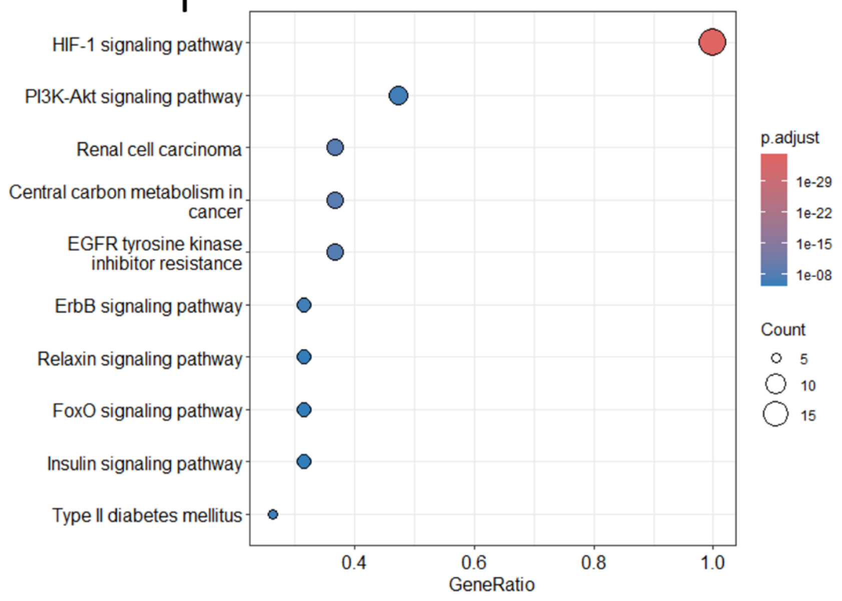

SUPPLEMENTARY FIGURE 4

A

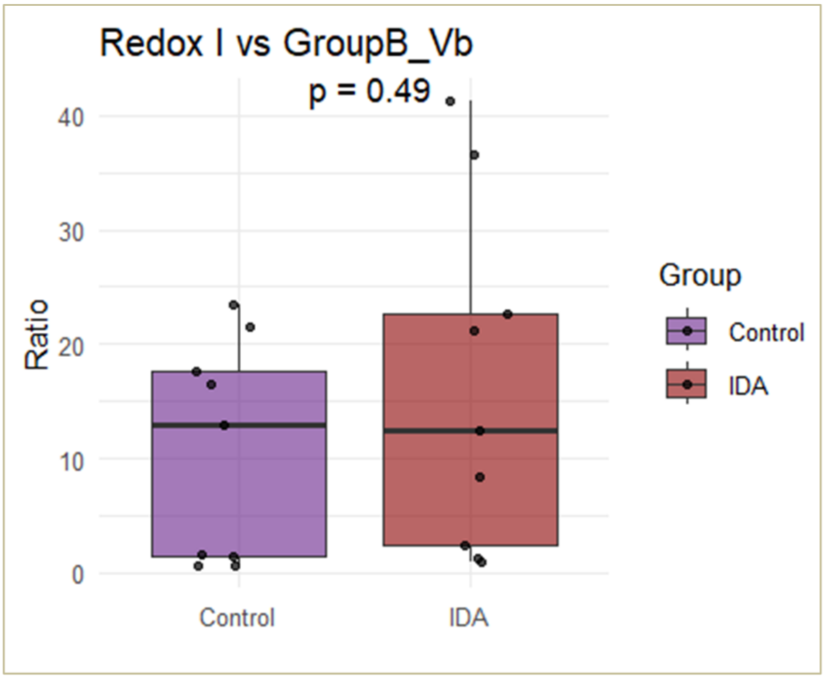

B

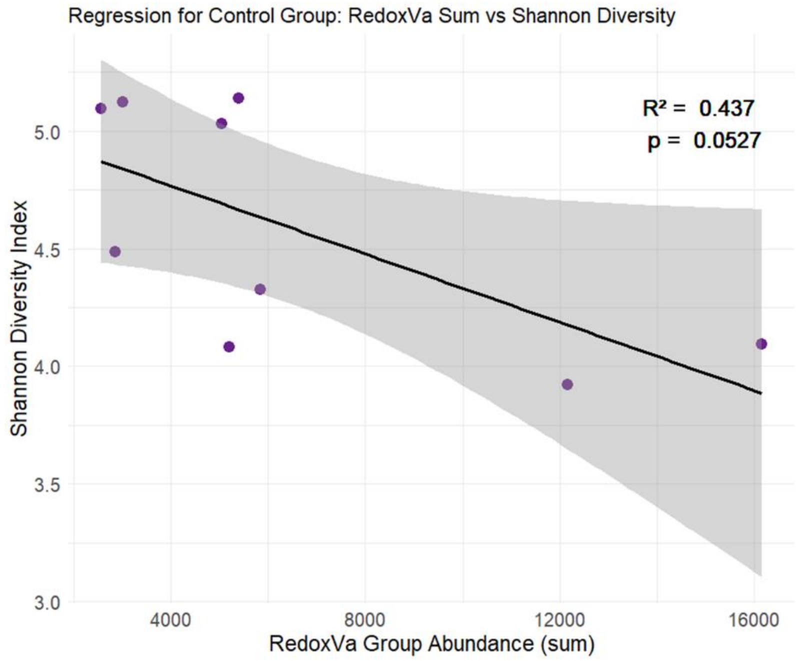

Regression for Control Group: RedoxVa Sum vs Shannon Diversity
R² = 0.437
p = 0.0527
Shannon Diversity Index
5.0
4.5
4.0
3.5
3.0
4000
8000
12000
16000
RedoxVa Group Abundance (sum)

C

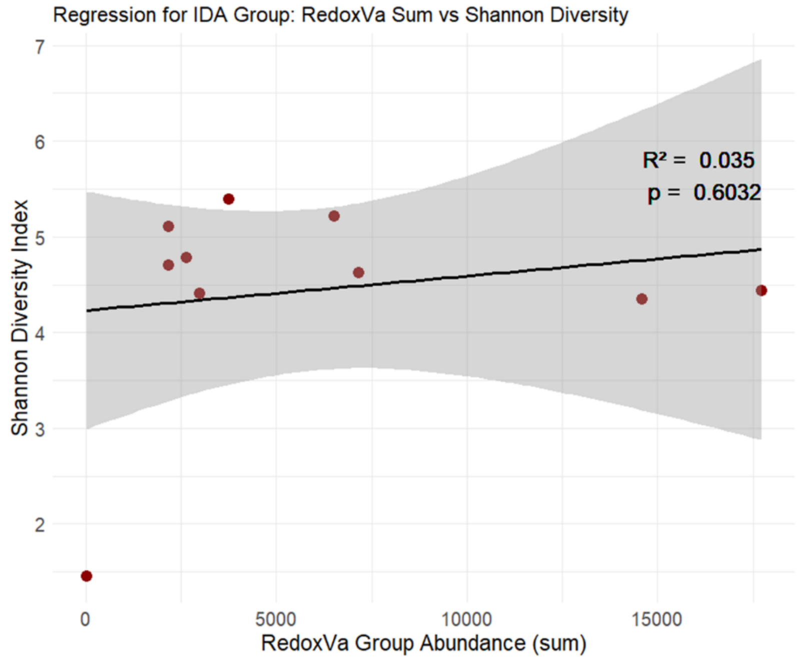

Regression for IDA Group: RedoxVa Sum vs Shannon Diversity
R² = 0.035
p = 0.6032
Shannon Diversity Index
7
6
5
4
3
2
0
5000
10000
15000
RedoxVa Group Abundance (sum)

D

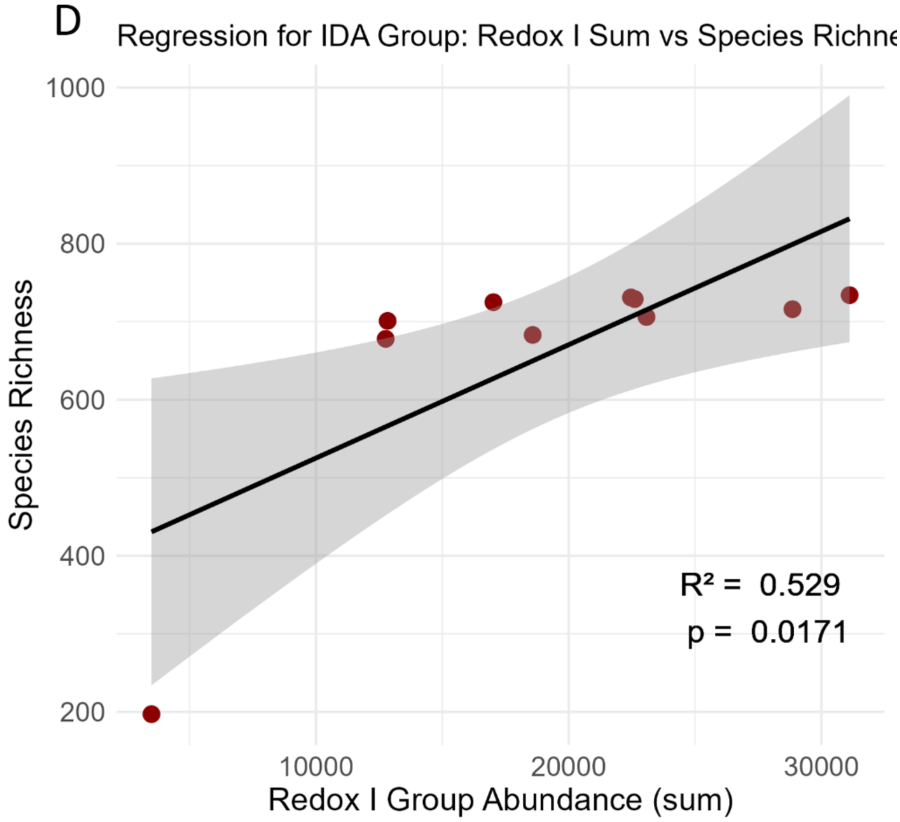

Regression for IDA Group: Redox I Sum vs Species Richne
1000
800
600
400
200
Species Richness
10000
20000
30000
Redox I Group Abundance (sum)
R² = 0.529
p = 0.0171

E

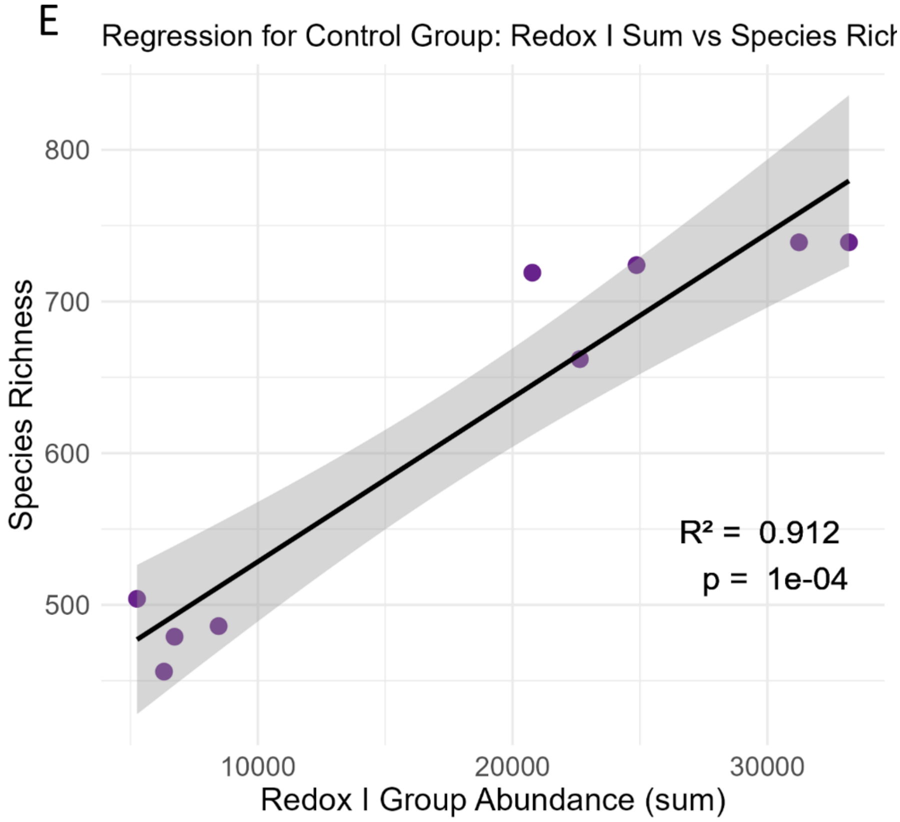

Regression for Control Group: Redox I Sum vs Species Rich
800
700
600
500
Species Richness
10000
20000
30000
Redox I Group Abundance (sum)
R² = 0.912
p = 1e-04

F

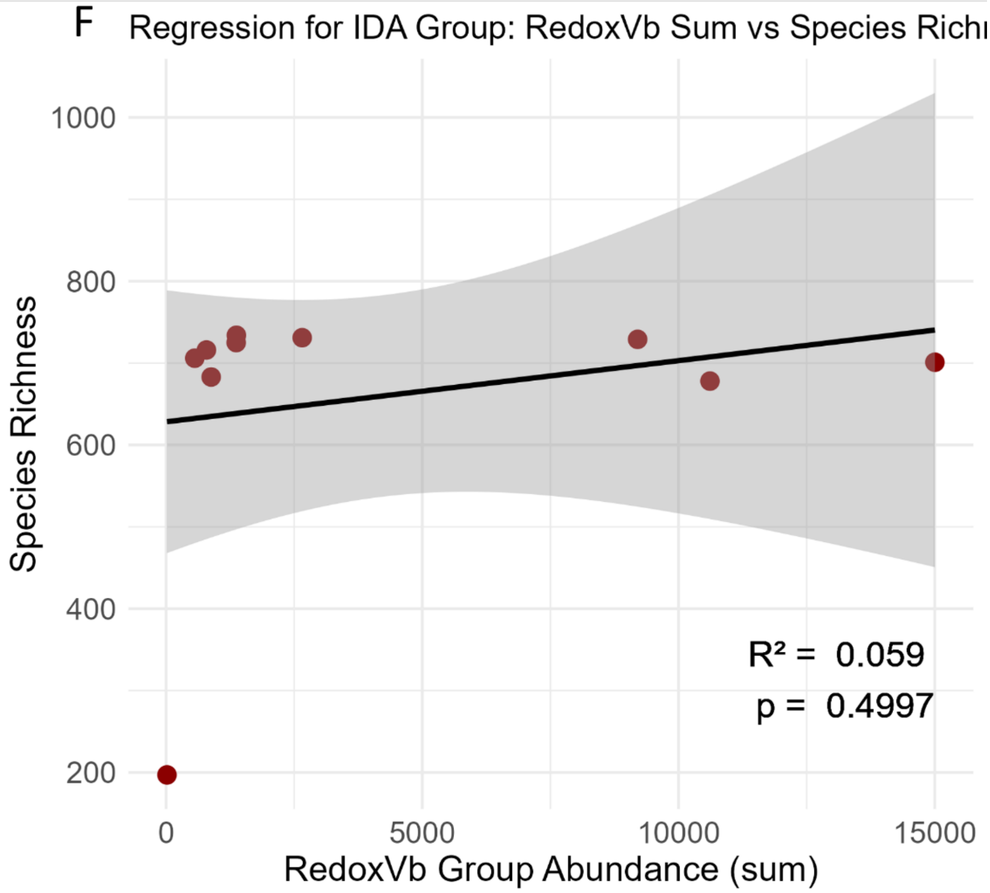
Regression for IDA Group: RedoxVb Sum vs Species Richı
Species Richness
1000
800
600
400
200
0
5000
10000
15000
RedoxVb Group Abundance (sum)
R² = 0.059
p = 0.4997

G

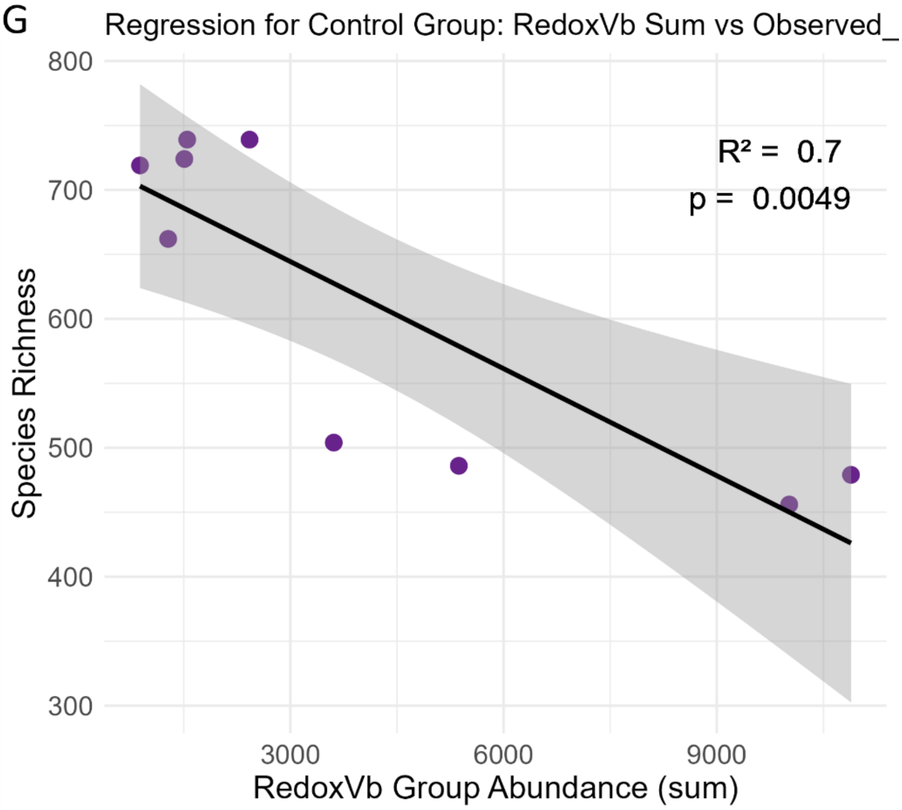
Regression for Control Group: RedoxVb Sum vs Observed_
Species Richness
800
700
600
500
400
300
3000
6000
9000
RedoxVb Group Abundance (sum)
R² = 0.7
p = 0.0049

SUPPLEMENTARY FIGURE 5

A

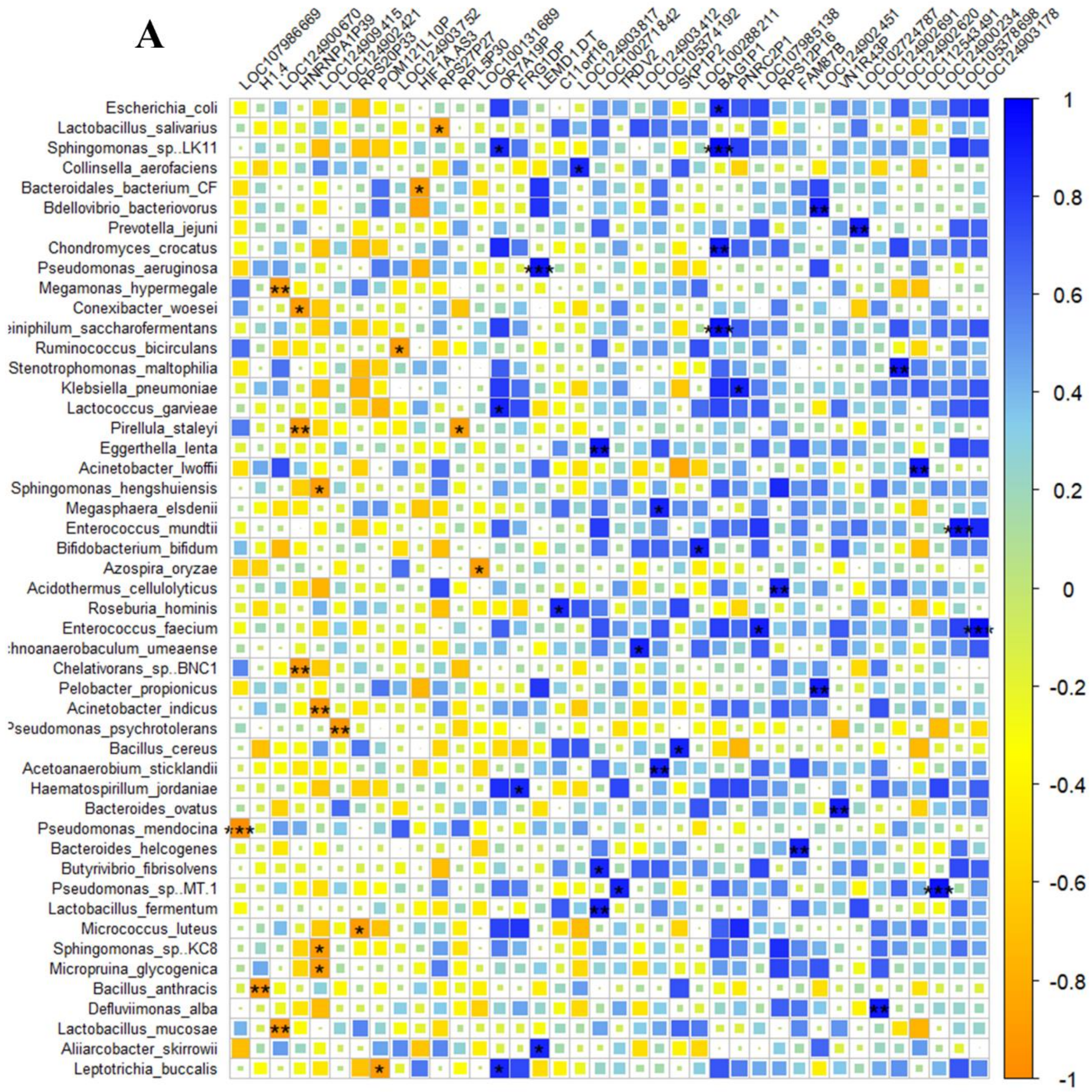

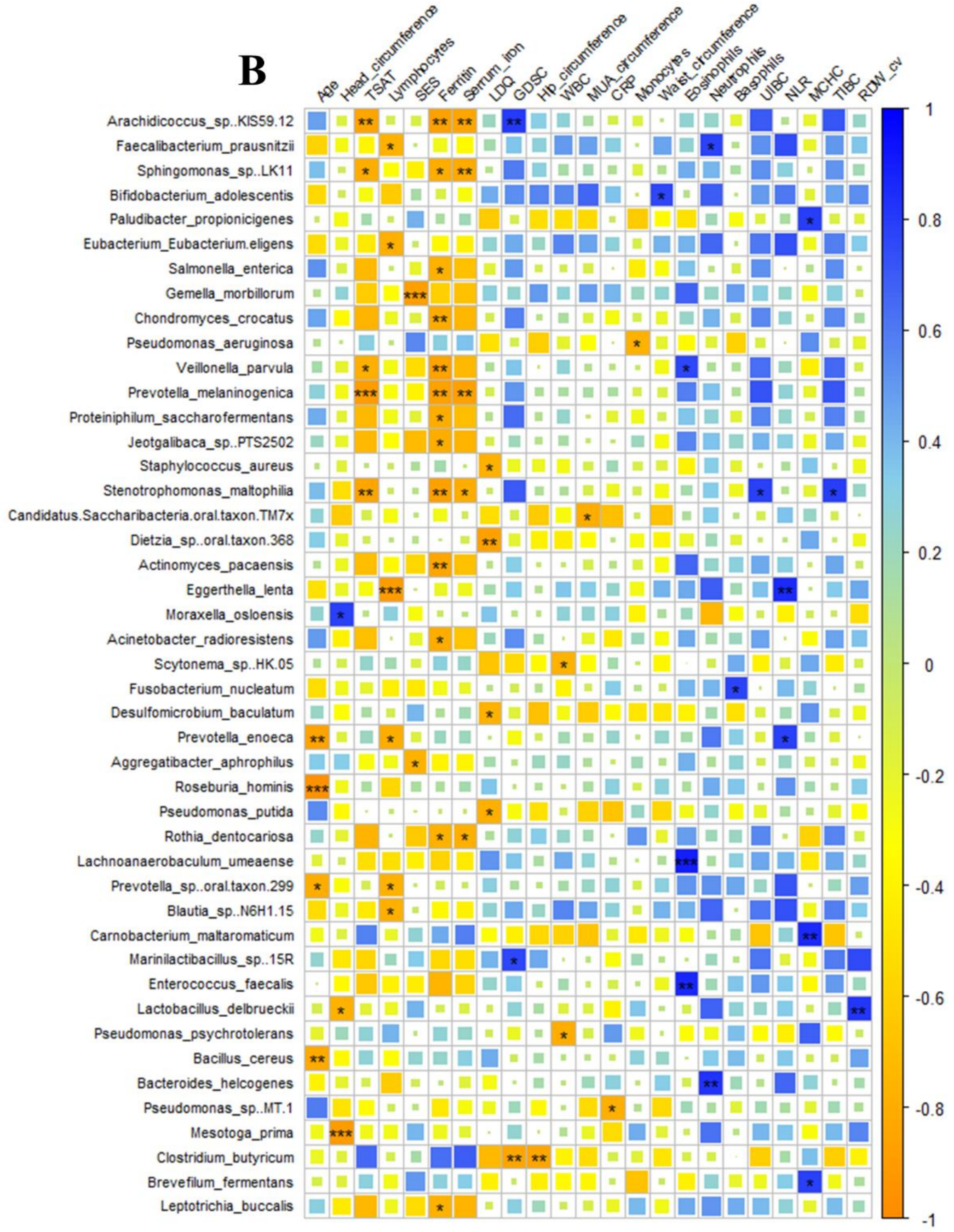
B
Age
Head_circumference
TSAT
Lymphocytes
SES
Ferritin
Serrum_iron
LDQ
GDSC
Hip_circumference
WBC
MUA_circumference
CRP
Monocytes
Waist_circumference
Eosinophils
Neutrophils
Basophils
UIBC
NLR
MCHC
TIBC
RDW_cv
Arachidicoccus_sp..KIS59.12
Faecalibacterium_prausnitzii
Sphingomonas_sp..LK11
Bifidobacterium_adolescentis
Paludibacter_propionicigenes
Eubacterium_Eubacterium.eligens
Salmonella_enterica
Gemella_morbillorum
Chondromyces_crocatus
Pseudomonas_aeruginosa
Veillonella_parvula
Prevotella_melaninogenica
Proteiniphilum_saccharofermentans
Jeotgalibaca_sp..PTS2502
Staphylococcus_aureus
Stenotrophomonas_maltophilia
Candidatus.Saccharibacteria.oral.taxon.TM7x
Dietzia_sp..oral.taxon.368
Actinomyces_pacaensis
Eggerthella_lenta
Moraxella_osloensis
Acinetobacter_radioresistens
Scytonema_sp..HK.05
Fusobacterium_nucleatum
Desulfomicrobium_baculatum
Prevotella_enoeca
Aggregatibacter_aphrophilus
Roseburia_hominis
Pseudomonas_putida
Rothia_dentocariosa
Lachnoanaerobaculum_umeaense
Prevotella_sp..oral.taxon.299
Blautia_sp..N6H1.15
Carnobacterium_maltaromaticum
Marinilactibacillus_sp..15R
Enterococcus_faecalis
Lactobacillus_delbrueckii
Pseudomonas_psychrotolerans
Bacillus_cereus
Bacteroides_helcogenes
Pseudomonas_sp..MT.1
Mesotoga_prima
Clostridium_butyricum
Brevefilum_fermentans
Leptotrichia_buccalis
1
0.8
0.6
0.4
0.2
0
-0.2
-0.4
-0.6
-0.8
-1

C

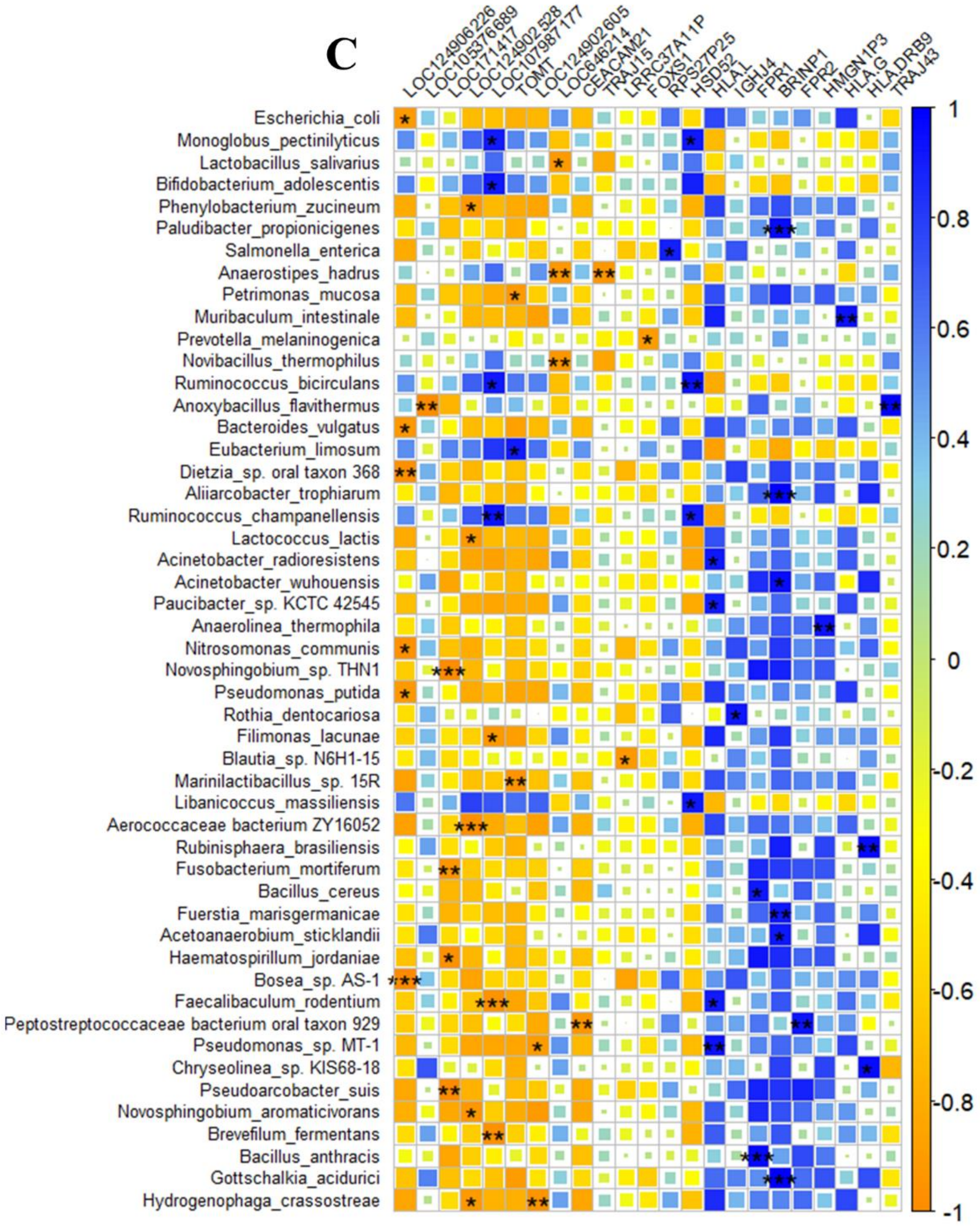

LOC124906226
LOC105376689
LOC171417
LOC124902528
LOC107987177
TOMT
LOC124902605
LOC646214
CEACAM21
TRAJ15
LRRC37A11P
FOXS1
RPS27P25
HSD52
HLA.L
IGHJ4
FPR1
BRINP1
FPR2
HMGN1P3
HLA.G
HLA.DRB9
TRAJ43
Escherichia_coli
Monoglobus_pectinilyticus
Lactobacillus_salivarius
Bifidobacterium_adolescentis
Phenylobacterium_zucineum
Paludibacter_propionicigenes
Salmonella_enterica
Anaerostipes_hadrus
Petrimonas_mucosa
Muribaculum_intestinale
Prevotella_melaninogenica
Novibacillus_thermophilus
Ruminococcus_bicirculans
Anoxybacillus_flavithermus
Bacteroides_vulgatus
Eubacterium_limosum
Dietzia_sp. oral taxon 368
Aliiarcobacter_trophiarum
Ruminococcus_champanellensis
Lactococcus_lactis
Acinetobacter_radioresistens
Acinetobacter_wuhouensis
Paucibacter_sp. KCTC 42545
Anaerolinea_thermophila
Nitrosomonas_communis
Novosphingobium_sp. THN1
Pseudomonas_putida
Rothia_dentocariosa
Filimonas_lacunae
Blautia_sp. N6H1-15
Marinilactibacillus_sp. 15R
Libanicoccus_massiliensis
Aerococcaceae bacterium ZY16052
Rubinisphaera_brasiliensis
Fusobacterium_mortiferum
Bacillus_cereus
Fuerstia_marisgermanicae
Acetoanaerobium_sticklandii
Haematospirillum_jordaniae
Bosea_sp. AS-1
Faecalibaculum_rodentium
Peptostreptococcaceae bacterium oral taxon 929
Pseudomonas_sp. MT-1
Chryseolinea_sp. KIS68-18
Pseudoarcobacter_suis
Novosphingobium_aromaticivorans
Brevefilum_fermentans
Bacillus_anthracis
Gottschalkia_acidurici
Hydrogenophaga_crassostreae
1
0.8
0.6
0.4
0.2
0
-0.2
-0.4
-0.6
-0.8
-1

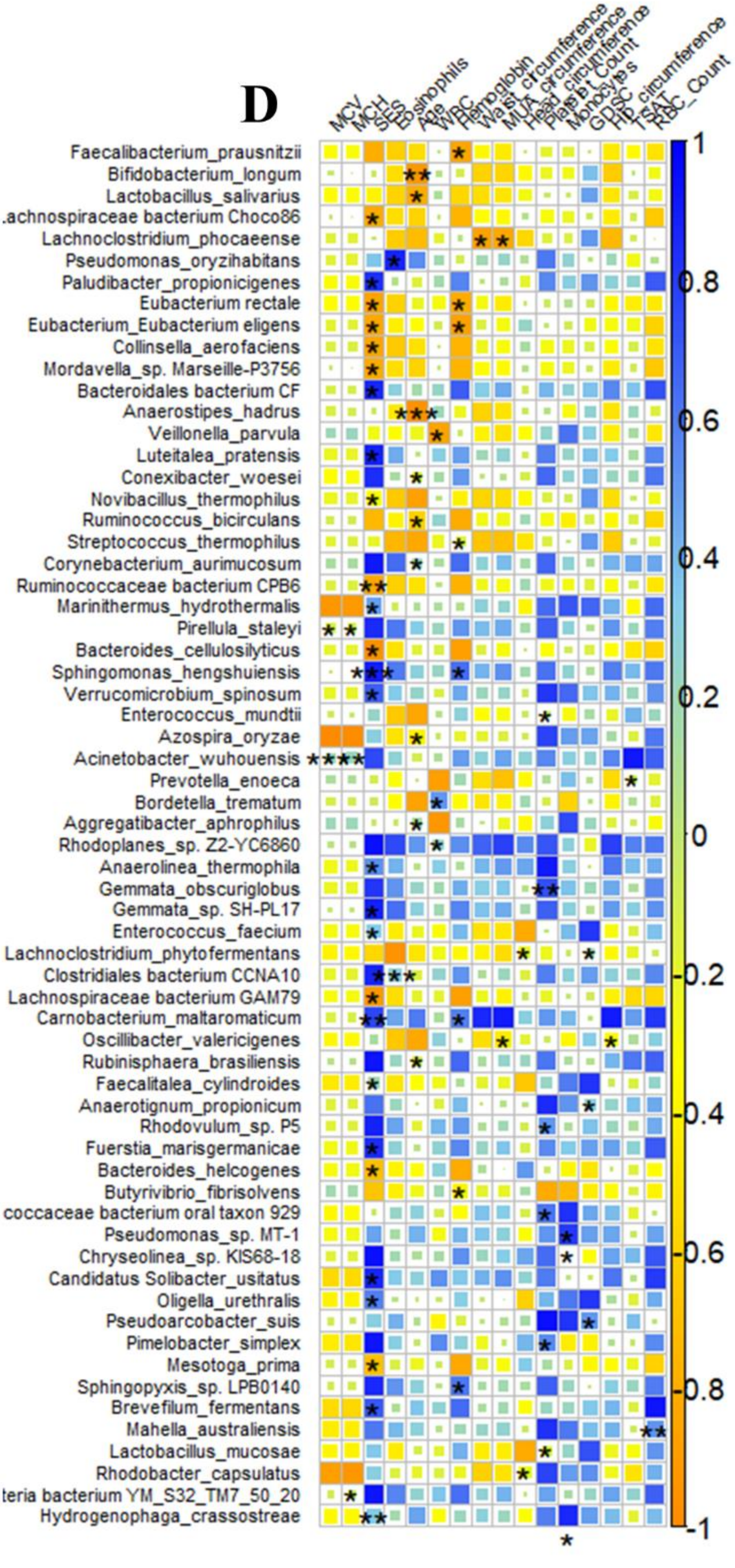
D
MCV
MCH
SES
Eosinophils
Age
WBC
Hemoglobin
Waist_circumference
MUA_circumference
Head_circumference
Platelet_Count
Monocytes
GDSC
HIP_circumference
TSAT
RBC_Count
Faecalibacterium_prausnitzii
Bifidobacterium_longum
Lactobacillus_salivarius
.achnospiraceae bacterium Choco86
Lachnoclostridium_phocaeense
Pseudomonas_oryzihabitans
Paludibacter_propionicigenes
Eubacterium rectale
Eubacterium_Eubacterium eligens
Collinsella_aerofaciens
Mordavella_sp. Marseille-P3756
Bacteroidales bacterium CF
Anaerostipes_hadrus
Veillonella_parvula
Luteitalea_pratensis
Conexibacter_woesei
Novibacillus_thermophilus
Ruminococcus_bicirculans
Streptococcus_thermophilus
Corynebacterium_aurimucosum
Ruminococcaceae bacterium CPB6
Marinithermus_hydrothermalis
Pirellula_staleyi
Bacteroides_cellulosilyticus
Sphingomonas_hengshuiensis
Verrucomicrobium_spinosum
Enterococcus_mundtii
Azospira_oryzae
Acinetobacter_wuhouensis
Prevotella_enoeca
Bordetella_trematum
Aggregatibacter_aphrophilus
Rhodoplanes_sp. Z2-YC6860
Anaerolinea_thermophila
Gemmata_obscuriglobus
Gemmata_sp. SH-PL17
Enterococcus_faecium
Lachnoclostridium_phytofermentans
Clostridiales bacterium CCNA10
Lachnospiraceae bacterium GAM79
Carnobacterium_maltaromaticum
Oscillibacter_valericigenes
Rubinisphaera_brasiliensis
Faecalitalea_cylindroides
Anaerotignum_propionicum
Rhodovulum_sp. P5
Fuerstia_marisgermanicae
Bacteroides_helcogenes
Butyrivibrio_fibrisolvens
coccaceae bacterium oral taxon 929
Pseudomonas_sp. MT-1
Chryseolinea_sp. KIS68-18
Candidatus Solibacter_usitatus
Oligella_urethralis
Pseudoarcobacter_suis
Pimelobacter_simplex
Mesotoga_prima
Sphingopyxis_sp. LPB0140
Brevefilum_fermentans
Mahella_australiensis
Lactobacillus_mucosae
Rhodobacter_capsulatus
teria bacterium YM_S32_TM7_50_20
Hydrogenophaga_crassostreae
1
0.8
0.6
0.4
0.2
0
-0.2
-0.4
-0.6
-0.8
-1
